# Visualization of direct and diffusion-assisted RAD51 nucleation by full-length human BRCA2 protein

**DOI:** 10.1016/j.molcel.2023.06.031

**Published:** 2023-07-26

**Authors:** Ondrej Belan, Luke Greenhough, Lucas Kuhlen, Roopesh Anand, Artur Kaczmarczyk, Dominika T. Gruszka, Hasan Yardimci, Xiaodong Zhang, David S. Rueda, Stephen C. West, Simon J. Boulton

**Affiliations:** 1 DSB Repair Metabolism Laboratory, The Francis Crick Institute, London NW1 1AT, UK; 2 DNA Recombination and Repair Laboratory, The Francis Crick Institute, London NW1 1AT, UK; 3 Section of Structural Biology, Faculty of Medicine, Imperial College London, Sir Alexander Fleming Building, South Kensington, London SW7 2AZ, UK; 4 Department of Infectious Disease, Faculty of Medicine, Imperial College London, London W12 0NN, UK; 5 Single Molecule Imaging Group, MRC-London Institute of Medical Sciences, London W12 0NN, UK; 6 Single Molecule Imaging of Genome Duplication and Maintenance Laboratory, The Francis Crick Institute, London NW1 1AT, UK

## Abstract

Homologous recombination (HR) is essential for error-free repair of DNA double-strand breaks, perturbed replication forks (RFs), and post-replicative single-stranded DNA (ssDNA) gaps. To initiate HR, the recombination mediator and tumor suppressor protein BRCA2 facilitates nucleation of RAD51 on ssDNA prior to stimulation of RAD51 filament growth by RAD51 paralogs. Although ssDNA binding by BRCA2 has been implicated in RAD51 nucleation, the function of double-stranded DNA (dsDNA) binding by BRCA2 remains unclear. Here, we exploit single-molecule (SM) imaging to visualize BRCA2-mediated RAD51 nucleation in real time using purified proteins. We report that BRCA2 nucleates and stabilizes RAD51 on ssDNA either directly or through an unappreciated diffusion-assisted delivery mechanism involving binding to and sliding along dsDNA, which requires the cooperative action of multiple dsDNA-binding modules in BRCA2. Collectively, our work reveals two distinct mechanisms of BRCA2-dependent RAD51 loading onto ssDNA, which we propose are critical for its diverse functions in maintaining genome stability and cancer suppression.

## Graphical abstract

**Graphical abstract:**
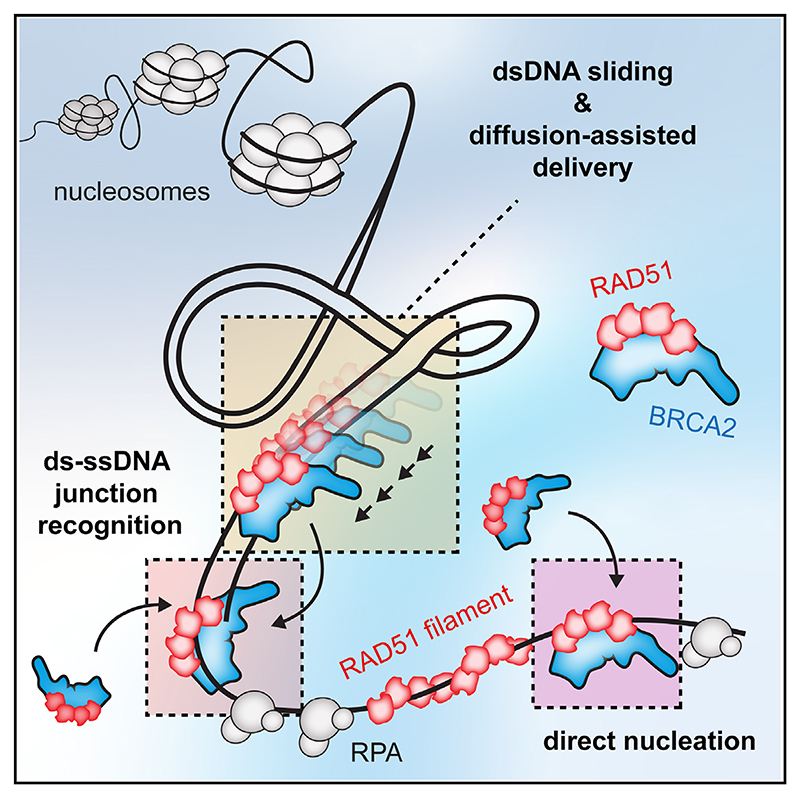

## Introduction

DNA double-strand breaks (DSBs) are toxic lesions that are a threat to genome stability. As such, unfaithful repair of DSBs can give rise to diseases such as cancer. Homologous recombination (HR) is largely an error-free pathway for the repair of DSBs, broken replication forks (RFs), and post-replicative single-stranded DNA (ssDNA) gaps operating in the S and G2 phases of the cell cycle.^1^ During HR, DSBs, or broken RFs, are nucleolytically resected to produce 3’ ssDNA overhangs, which are tightly bound by replication protein A (RPA). For HR to occur, RPA must be replaced by the RAD51 recombinase in the form of a helical nucleoprotein filament, which is capable of homology search within the sister chromatid or homologous chromosome. Strand invasion of the 3’ end of ssDNA with the intact template results in the formation of a displacement loop (D-loop) product, which is then processed by multiple redundant pathways to repair the broken DNA.

RAD51 has lower affinity for ssDNA compared with RPA and cannot outcompete RPA under physiological conditions. Hence, recombination mediators have evolved to facilitate RAD51 filament assembly. Among the most well-established recombination mediator proteins is the breast cancer susceptibility protein 2 (BRCA2). Human BRCA2 is a large, 3,418 amino acid protein that contains multiple domains. Among these are eight BRC repeats that bind RAD51,^2–4^ a C-terminal DNA-binding domain (C-DBD, residues 2,474–3,190) composed of three tandem oligosaccharide-binding (OB) folds and a tower domain,^5^ and a C-terminal RAD51 interaction site within the region encoded by exon 27, referred to as the TR2 domain (residues 3,265–3,330).^6,7^ Recent work has suggested that region within exon 27 of BRCA2 also harbors a second DNA-binding site, as evident by ssDNA and double-stranded DNA (dsDNA) binding activity of isolated 3,194–3,418 C-terminal fragment of BRCA2. We refer to this potential DNA-binding domain as the extreme C-terminal DNA-binding region (exC-DBR; residues 3,194–3,418).^8^ In addition, similar to its *Ustilago maydis* ortholog, Brh2,^9^ human BRCA2 also contains an N-terminal DNA-binding domain (N-DBD, residues 250–500) related to the zinc-finger domain of PARP1 (poly [ADP-ribose] polymerase 1).^10^

BRCA2 has also been implicated in the import of RAD51 into the nucleus,^4^ initial RPA displacement,^11^ nucleation of RAD51 on ssDNA^12–14^ and stabilization of RAD51 filaments.^3,6,7,15^ RAD51 filaments form in two steps: rate-limiting nucleation and subsequent filament growth.^16^ Using purified human proteins and negative stain electron microscopy (EM)^17^ and real-time single-molecule (SM) imaging of nematode RAD-51 and the BRCA2 homolog (BRC-2),^16^ it was established that BRCA2 stimulates predominantly RAD51 nucleation rather than filament growth. The crystal structure of BRC repeat bound to RAD51 monomer suggested that BRCA2 would contact RAD51 at the 3’ end of the filament.^18^ Furthermore, full-length (fl) BRCA2 was found at the 3’ ends of RAD51 filaments by negative stain EM.^17^ Although high-resolution structures of fl BRCA2 have not been reported, low-resolution negative stain EM images suggest that BRCA2 forms predominantly monomeric^19^ and dimeric^17^ species. Other reports using AFM observed multimerization of fl BRCA2 into heterogeneous large molecular assemblies.^20^

Apart from its well-established role in DSB repair, BRCA2 was shown to have a role during DNA replication. BRCA2 was shown to stabilize RAD51 at RFs to protect nascent strands from MRE11-dependent nucleolytic degradation,^21^ which occurs particularly after RF reversal.^22^ BRCA2, together with BRCA1, was also shown to suppress post-replicative ssDNA gap formation following PARP inhibitor treatment.^23^

Although BRCA2 was first proposed to interact with dsDNA over 20 years ago when the crystal structure of the C-terminal DBD was reported,^5^ the duplex binding capability of BRCA2 has been largely unexplored.^3,12,14^ By employing SM imaging techniques to directly visualize RAD51 nucleation by fl human BRCA2, we show that BRCA2 delivers RAD51 to DNA, promotes its nucleation onto ssDNA, and subsequently stabilizes ssDNA-bound RAD51 clusters. Unexpectedly, we show that BRCA2 is also capable of binding and diffusing along the dsDNA back-bone, with BRCA2, and therefore RAD51 accumulating preferentially at the two ss-dsDNA junctions in the presence of RPA, with higher preference for the 5’ junction. We demonstrate that BRCA2 moves predominantly through sliding on dsDNA but is constrained and cannot circumvent nucleosomes. Finally, we dissect the relative contribution of the dsDNA-binding domains of BRCA2 to this process, which reveals the cooperative involvement of several DNA-binding modules. Collectively, these results shed light on the molecular mechanism of the early steps of RAD51 filament establishment and provide unexpected insights into the intrinsic versatility and modular nature of BRCA2 as a RAD51 loader during its role in DSB repair, RF protection, and post-replicative ssDNA gap suppression.

## Results

### Human BRCA2 nucleates and stabilizes RAD51 clusters on ssDNA

**Figure 1:**
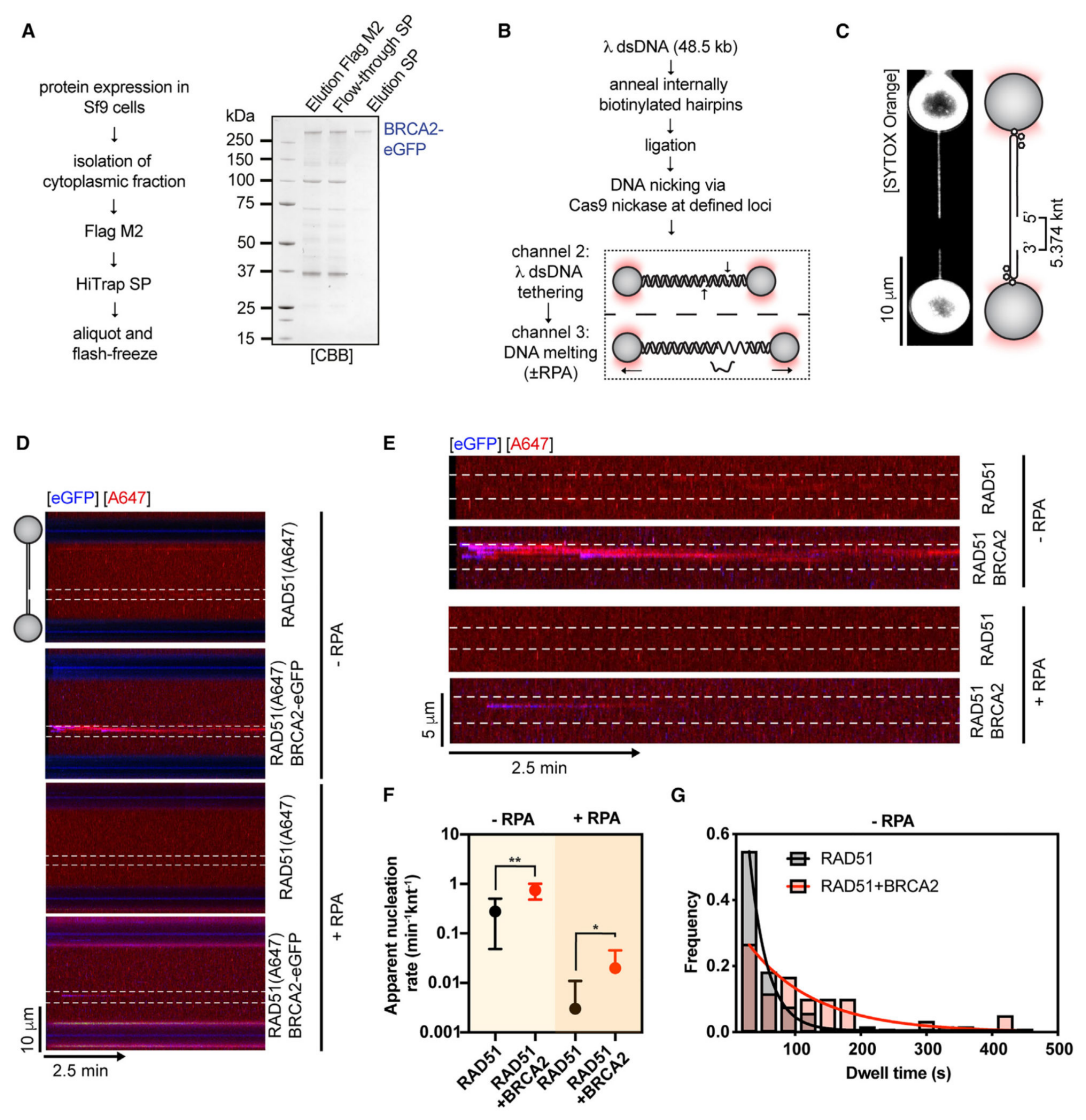
Single-molecule imaging of RAD51 nucleation by full-length human BRCA2 (A) Purification of full-length human BRCA2-eGFP. (B) Schematic of the protocol used to generate gapped λ DNA (gDNA) substrates. (C) Representative image of an asymmetrically positioned ssDNA gap within λ DNA held at 10 pN force. (D) Kymograph showing the binding of 25 nM RAD51(A647) (red) to gDNA in the presence/absence of 5 nM BRCA2-eGFP (blue) and/or 1.25 nM RPA in the presence of 5 nM SYTOX Orange, 100 mM NaCl, 2 mM MgCl_2_, 1 mM CaCl_2_, and 2 mM ATP at ~5 pN force. Position of the ssDNA gap is indicated by dashed lines. (E) Zoom in on the ssDNA gap region from kymographs in (D). (F) Quantification of apparent RAD51(A647) nucleation rates in the indicated conditions (n = 5–21 molecules). Dots represent mean. Error bars represent SEM. p values by Student’s t test. n.s., p > 0.05; *p ≤ 0.05; **p ≤ 0.01. (G) Histograms of dwell times of RAD-51(A647) in the absence (N = 104 clusters) or presence of BRCA2-eGFP (N = 62 clusters). Lines represent exponential fits. See also Figure S1.

To reconstitute RAD51 nucleation on RPA-coated ssDNA at the SM level, we purified fl human BRCA2 from baculovirus-infected insect cells (Figure S1A). BRCA2 was then fused at the C terminus to monomeric eGFP, expressed, and purified for SM imaging (Figures 1A and S1B). We verified that BRCA2 purified in this manner retains high ssDNA affinity (Figure S1C) and the ability to load RAD51 onto RPA-coated ssDNA in ensemble DNA capture assays (Figure S1D). RAD51^C319S^ was fluorescently labeled by an Alexa Fluor 647 C2 maleimide ester, as described previously.^24–26^ We previously confirmed that labeled RAD51 largely retains its activity *in vitro*,^24^ and we refer to it as RAD51(A647) in the text. We then used a combination of optical tweezers, confocal fluorescence microscopy, and microfluidics (C-trap setup)^16,24^ (Figure 1B) to directly visualize BRCA2-mediated RAD51 nucleation on 5.374 knt-long ssDNA gaps asymmetrically positioned within ~ 48 kb long λ dsDNA^27^ (Figure 1C). As initially shown from previous bulk observations,^12–14^ addition of 5 nM BRCA2-eGFP to 25 nM RAD51(A647) resulted in an ~2.7-fold increase in the mean nucleation rate compared with RAD51(A647) alone (Figures 1D–1F).

Next, we performed nucleation experiments in the presence of 1.25 nM RPA to reflect the physiological substrate for RAD51 assembly in cells. When compared with conditions without RPA (0.277 knt^−1^min^−1^), we observed strong suppression of spontaneous RAD51(A647) nucleation frequencies in the presence of RPA (0.003 knt^−1^min^−1^). Even though overall nucleation levels are low in the presence of RPA, the addition of 5 nM BRCA2-eGFP increased nucleation rates ~6.6-fold (Figure 1F). Taken together, these data indicate that BRCA2 serves as a universal RAD51 loading factor that becomes critical in the presence of the ssDNA-binding competitor, RPA. We were also able to monitor a co-localized eGFP fluorescent signal, confirming that BRCA2-eGFP is present on ssDNA during RAD51(A647) nucleation. Next, we analyzed dwell times of RAD51 clusters, which increased from τ ~ 27.2 s to τ ~ 101.9 s upon addition of BRCA2-eGFP, indicating that BRCA2 also stabilizes RAD51 nuclei on ssDNA (Figure 1G).

Due to fast photobleaching of the BRCA2-eGFP signal during nucleation experiments, we were unable to accurately estimate BRCA2 dwell times. However, we were able to perform single-step photobleaching analysis^28,29^ to estimate the number of BRCA2-eGFP molecules in individual diffraction-limited nucleation events. BRCA2 nucleates RAD51 as a heterogeneous complex, with BRCA2 monomers and dimers being the predominant species (Figures S1E and S1F). This is consistent with the polydisperse profile of our BRCA2 preparations on negative stain EM grids (Figure S1G), native polyacrylamide gel electrophoresis of fl BRCA2 (Figure S1H), and data from published AFM^20^ and EM studies.^17^ We note that due to the large number of Alexa Fluor 647 fluorophores in BRCA2-RAD51 nucleation clusters, we were unable to accurately estimate the number of photobleaching steps for RAD51(A647).^28^

### BRCA2-RAD51 nucleation complex accumulates at ds-ssDNA junctions in the presence of RPA

**Figure 2:**
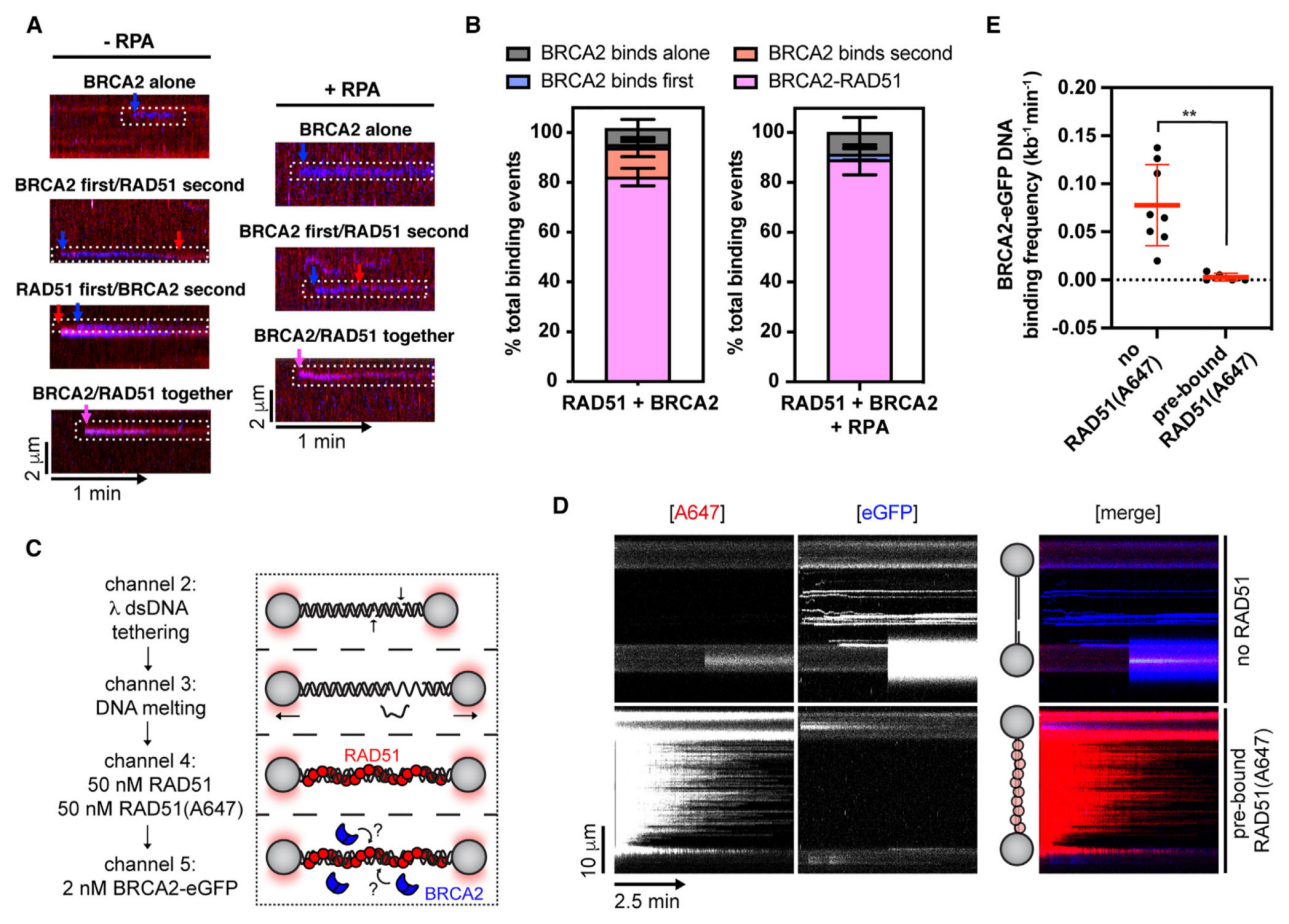
BRCA2 and RAD51 bind to DNA as a complex (A) Examples of different orders of binding events observed for BRCA2-eGFP and RAD51(A647). (B) Quantification of different BRCA2-eGFP/RAD51(A647) binding events in the absence (n = 7 molecules, N = 76 events) or presence (n = 9 molecules, N = 29 events) of 1.25 nM RPA. Error bars represent SEM. (C) Schematic of RAD51 filament binding experiment, in which λ gDNA was pre-incubated with a 1:1 mixture of labeled and unlabeled RAD51 and then moved to a channel containing BRCA2-eGFP to monitor BRCA2 binding. (D) Kymographs showing the binding of 2 BRCA2-eGFP (blue) to gDNA or gDNA pre-coated with 50 nM RAD51 and 50 nM RAD51(A647) in the presence of 100 mM NaCl, 2 mM MgCl_2_, 1 mM CaCl_2_, and 2 mM ATP held at ~5 pN force. Position of the ssDNA gap is indicated. (E)Quantification of BRCA2 binding frequencies on λ gDNA or λ gDNA pre-coated with RAD51. Lines represent mean. Error bars represent SD. p values by Student’s t test. n.s., p > 0.05; *p ≤ 0.05; **p ≤ 0.01. See also Figure S2.

To better understand how the BRCA2-RAD51-ssDNA complex forms, we analyzed individual BRCA2-RAD51 binding events. We categorized the events based on the order of BRCA2 and RAD51 binding, which included the following: (1) BRCA2-RAD51 binds as a co-complex in a single step, (2) BRCA2 binds to ssDNA first and then recruits RAD51 from solution, (3) BRCA2 binds pre-formed ssDNA-bound RAD51 clusters, or (4) BRCA2 binds alone without any detectable RAD51(A647) signal (Figure 2A). We observed that in ~80% of binding events, BRCA2-RAD51 binds as a co-complex in a single step in the presence or absence of RPA (Figure 2B). These findings are consistent with observations that most of nuclear RAD51 is bound to BRCA2 in cells^4,30^ and displays mobility similar to that of BRCA2 following DNA damage.^30^

Previous work using short BRCA2 peptides that bind RAD51 suggested that BRCA2 may bind laterally to and stabilize RAD51 filaments.^6,7^ However, fl BRCA2 was observed predominantly at filament ends by negative stain EM.^17^ Although our data suggest that RAD51 filament recognition by BRCA2 is a minor event, the sparse RAD51 nucleation might bias the analysis. To circumvent this possibility, we pre-incubated gDNA substrates with a 1:1 ratio of unlabeled RAD51 and RAD51(A647) (Figure 2C). Extensive RAD51 filament assembly was evident from the characteristic change in the DNA force-distance curve^31^ and strong red fluorescence signal uniformly distributed along the gDNA substrate (Figure S2A). Pre-assembled RAD51 filaments were then moved to a channel containing BRCA2-eGFP to monitor its recruitment (Figure 2D). Strikingly, we observed that pre-bound RAD51 almost completely excluded BRCA2-eGFP binding (Figures 2D and 2E), indicating that BRCA2 does not significantly associate with pre-assembled RAD51 filaments, supporting previous EM data^17^ and the notion that BRCA2 associates with RAD51 in solution and only during nucleation.

**Figure 3:**
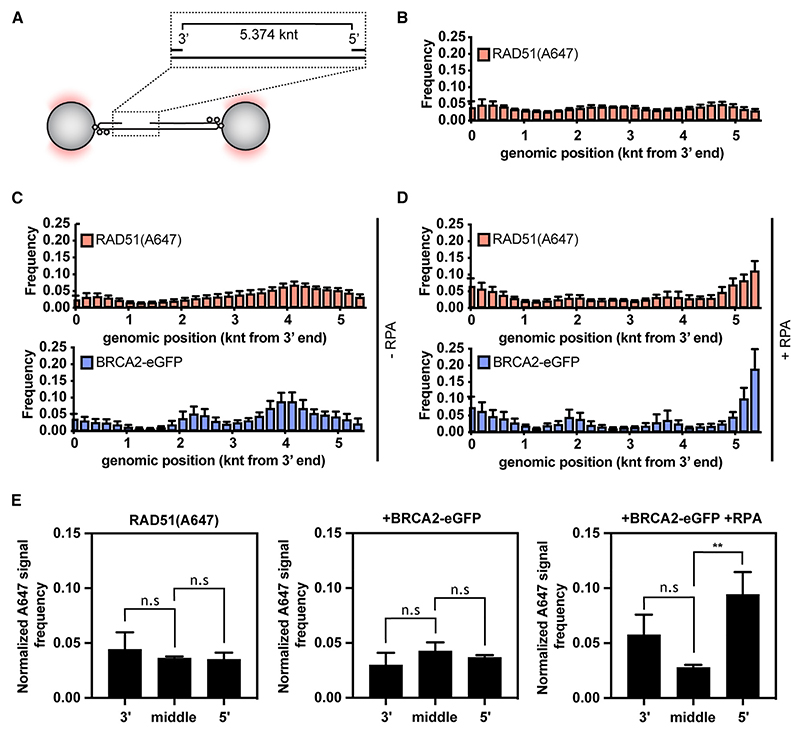
BRCA2 nucleates RAD51 at the edges of ssDNA gap in the presence of RPA (A) Schematic of 5.374 knt-long ssDNA embedded within λ dsDNA. (B) Positional analysis of RAD51(A647) (red) binding along the length of 5.374 knt ssDNA gap. 200 nt bins. n = 5 molecules. Error bars represent SEM. 200 nt bins. (C) Position analysis of RAD51(A647) (red) or BRCA2-eGFP (blue) binding along the length of 5.374 knt ssDNA gap in the absence of RPA. n = 6 molecules. Error bars represent SEM. 200 nt bins. (D) Position analysis of RAD51(A647) (red) or BRCA2-eGFP (blue) binding along the length of 5.374 knt ssDNA gap in the presence of 1.25 nM RPA. n = 11 molecules. Error bars represent SEM. 200 nt bins. (E) Per-bin normalized A647 signal frequency for 3’ and 5’ ds-ssDNA junction (3 and 3 bins) or middle of the ssDNA gap (21 bins) in the indicated conditions. Error bars represent SEM. p values by Student’s t test. n.s., p > 0.05; *p ≤ 0.05; **p ≤ 0.01. See also Figure S2.

Initial studies of the BRCA2 homolog from *Ustilago maydis*, Brh2, suggested recruitment to 5’ ds-ssDNA junctions, from which RAD51 filaments subsequently grow.^32^ This was explained by preferential binding of Brh2 to 5’ ds-ssDNA junctions when compared with 3’ ds-ssDNA junctions, dsDNA and ssDNA. However, human BRCA2 does not display strong ds-ssDNA junction preference, at least in the absence of RPA.^14^ When RPA is present, BRCA2 stimulates RAD51-mediated DNA strand exchange reactions more strongly with substrates containing either 3’ or 5’ ds-ssDNA junctions.^12^ In our experiments, the stretched gDNA substrate contained both 3’ and 5’ ds-ssDNA junctions separated by 5,374 nucleotides of ssDNA (Figure 3A). This allowed us to monitor the recruitment of BRCA2-eGFP along the gDNA. In accordance with previous studies,^33^ RAD51 or BRCA2/RAD51 complexes bound largely uniformly along the length of ssDNA gaps (Figure 3B). The presence of BRCA2-eGFP correlated well with the presence of RAD51 (A647), consistent with our previous observations that the two proteins bind ssDNA as a co-complex (Figure 3C). However, in the presence of RPA, we observed a shift of BRCA2-eGFP and RAD51(A647) signals to the edges of ssDNA gaps (Figure 3D), indicating enrichment at 3’ and 5’ ds-ssDNA junctions, although there is a statistically significant preference for 5’ ds-ssDNA junction (Figure 3E), consistent with the reported subtle preference for substrates containing 5’ ds-ssDNA junction at high salt concentrations.^12^ We also note that in the absence of RPA, BRCA2/RAD51 complexes accumulate asymmetrically within the ssDNA gap (Figure 3C) in a manner that correlates with GC-content of the underlying ssDNA (Figure S2B). Although some OB-fold-containing proteins show preference for GC-rich sequences, such as the CST complex binding to telomeric DNA,^34^ this phenomenon could also be explained by a few short fragments of the melted DNA remaining annealed to GC-regions of the gap in the absence of RPA, creating an internal ds-ssDNA junction potentially recognized by BRCA2.

### BRCA2-RAD51 complex diffuses along dsDNA backbone

**Figure 4:**
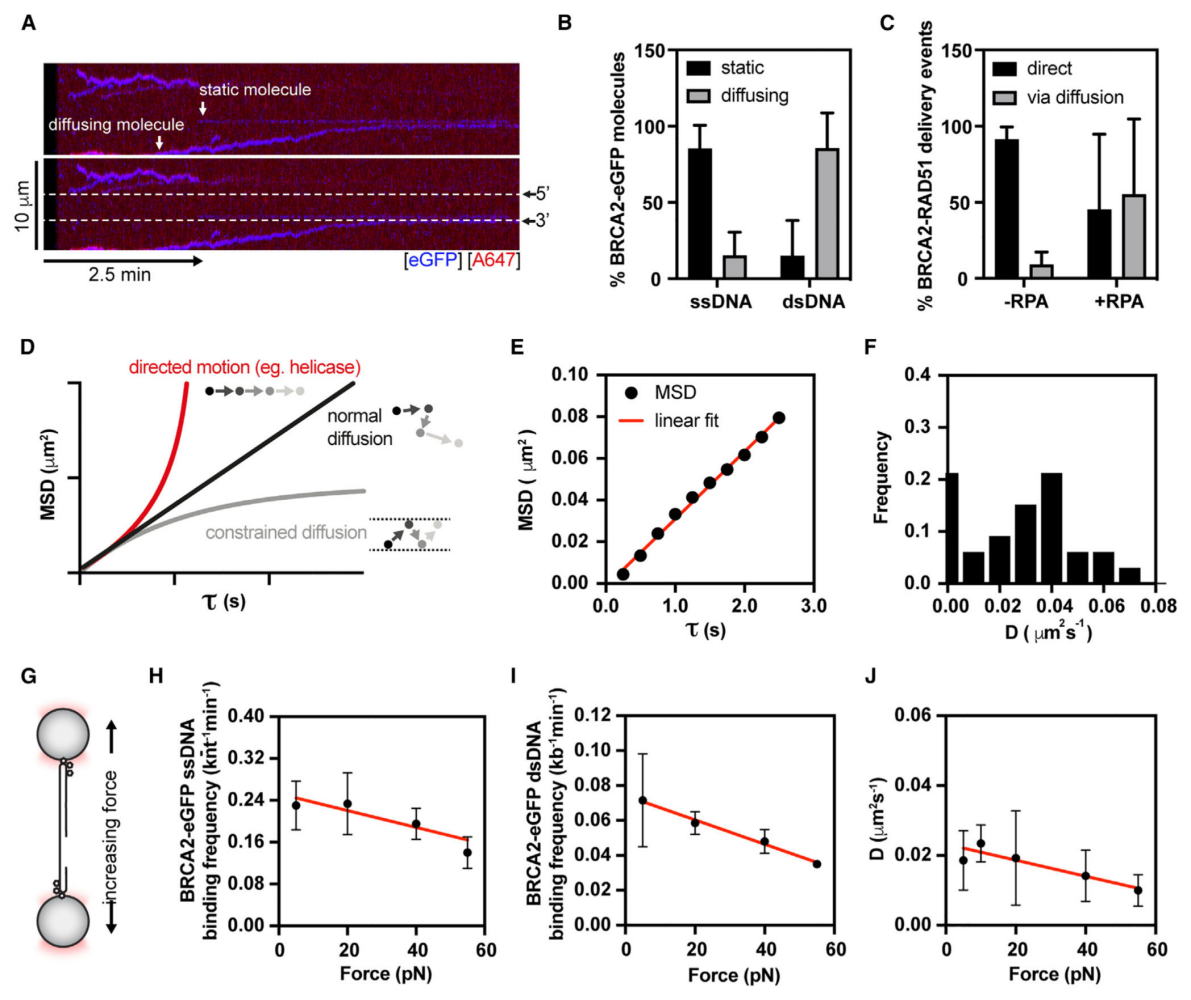
BRCA2 moves along dsDNA arms via normal diffusion (A) A representative kymograph showing diffusion-driven delivery of BRCA2-RAD51 complexes to ssDNA in the vicinity of the ds-ssDNA junction. Static BRCA2-eGFP molecules bound directly to the ssDNA gap or mobile BRCA2-eGFP molecules diffusing along dsDNA are indicated by arrows. 25 nM RAD51(A466) (red) was incubated with gDNA in the presence of 5 nM BRCA2-eGFP (blue) and 1.25 nM RPA in the presence of 5 nM SYTOX Orange. Position of the ssDNA gap is indicated by dashed lines. (B) Fraction of diffusive or static BRCA2-eGFP molecules on ss-(n = 9) or dsDNA (n = 15). Error bars represent SD. (C) Relative contribution of diffusion-assisted RAD51 delivery to RAD51 accumulation at the ssDNA gap in the presence (n = 15) or absence of RPA (n = 10). Error bars represent SD. (D) Mean square displacement (MSD) as a function of tau to estimate the mode of particle movement from the shape of the curve. (E) An example of MSD analysis of a moving BRCA2-eGFP molecule on dsDNA showing a linear relationship/normal diffusion. (F) Diffusion coefficient (D) calculated from MSD analysis for BRCA2-RAD51 complexes moving on dsDNA. 2 nM BRCA2-eGFP, 20 nM RAD51(A647), 10 pN force, 25 mM NaCl, 2 mM MgCl_2_, and 2 mM ATP. Bin size = 0.01 mm^2^s^−1^. N = 33 molecules. (G) Schematic of the force-pulling experiment, where the position of an optical trap is changed to exert tension on gDNA. (H) ssDNA-binding frequency of BRCA2-eGFP as a function of force. 2 nM BRCA2-eGFP, 20 nM RAD51(A647). 100 mM NaCl, 2 mM MgCl_2_, 1 mM CaCl_2_, and 2 mM ATP. Red line represents linear regression. Error bars represent SEM. n = 2–6 molecules. (I) dsDNA-binding frequency of BRCA2-eGFP as a function of force. 2 nM BRCA2-eGFP, 20 nM RAD51(A647). 100 mM NaCl. Red line represents linear regression. Error bars represent SEM. n = 3–6 molecules. (J) Diffusion coefficient of BRCA2-eGFP as a function of force. 2 nM BRCA2-eGFP, 20 nM RAD51(A647). 50 mM NaCl, 2 mM MgCl_2_, and 2 mM ATP. Red line represents linear regression. Error bars represent SEM. N = 36–50 molecules. See also Figure S3.

Upon detailed inspection of the BRCA2 recruitment kymographs, we noted that in addition to direct binding within the ssDNA gap, we also observed RAD51-BRCA2 molecules moving along dsDNA arms that frequently stopped once they reached the 3’ or 5’ ds-ssDNA junctions (Figure 4A). Although fl BRCA2 was reported to have a low affinity for short dsDNA substrates^12,14^ (K_D_ > 200 nM), we were able to observe binding to and movement along dsDNA at nanomolar BRCA2 concentrations. Recently, other proteins, such as endonuclease I, were observed to bind and diffuse on dsDNA despite apparent low dsDNA affinity.^35^ BRCA2-RAD51 complexes move mostly on dsDNA, whereas they display static behavior on ssDNA (Figure 4B). In the presence of RPA, slightly less than half of RAD51 nucleation events at the ssDNA gaps result from RAD51-BRCA2 molecules that reach the ssDNA gap by moving along the dsDNA arms (Figure 4C). Importantly, BRCA2 inclusion does not lead to extensive filamentation on dsDNA, reflecting its ability to target RAD51 specifically to ssDNA.

To understand the movement of BRCA2 on dsDNA in more detail, we extracted the trajectory of fluorescent molecules using a custom-written single-particle tracking algorithm^35^ and then calculated the mean square displacement (MSD) of moving BRCA2-RAD51 complexes. Proteins can move on DNA via several mechanisms, including free 1D diffusion (random walk), constrained diffusion (where diffusion barriers are present), and directed motion (e.g., ATPase-driven translocation). This information can be readily extracted from the shape of the MSD-tau plot (Figure 4D). For BRCA2-RAD51 particles, we found that the MSD increases linearly with the time interval (tau), indicating that BRCA2-RAD51 complexes move on the dsDNA via 1D diffusion (Figure 4E). From the slope of the MSD curve, we calculated the diffusion coefficient, D, for individual BRCA2/RAD51 complexes, with a mean value of 0.040 ± 0.014 (95% CI) μm^2^s^−1^ (Figure 4F). These values are comparable to D values obtained for dsDNA diffusion of the similar-sized SWR1 complex.^36^ We also observed that BRCA2 exists in two populations: slow-moving molecules (first bin) and fast-moving diffusing molecules. The presence of RAD51 does not significantly impact BRCA2 diffusion rates (Figures S3A and S3B), indicating that the diffusion of BRCA2-RAD51 complex on dsDNA is mediated primarily by the DNA-binding domains of BRCA2 and that RAD51 does not largely affect the diffusion of BRCA2 complexes due to its low molecular weight relative to BRCA2. The presence of RAD51 results in a modest, albeit statistically non-significant, decrease in the overall DNA-binding frequencies of BRCA2 (Figures S3A and S3C). The wide distribution of D in our datasets is likely due to the heterogeneous nature of BRCA2 oligomers.

Next, we asked whether DNA tension influences the diffusion parameters of BRCA2-RAD51 particles (Figure 4G), such as in the case of Cas12a, where increasing force dramatically increases the diffusion coefficient.^37^ In contrast to Cas12a, BRCA2-eGFP ssDNA and dsDNA binding is largely force-independent with a modest decrease in binding frequency (Figures 4H and 4I) and diffusion coefficient (Figure 4J) observable at very high forces (~50 pN).

### BRCA2-RAD51 complex diffuses via sliding on dsDNA

**Figure 5:**
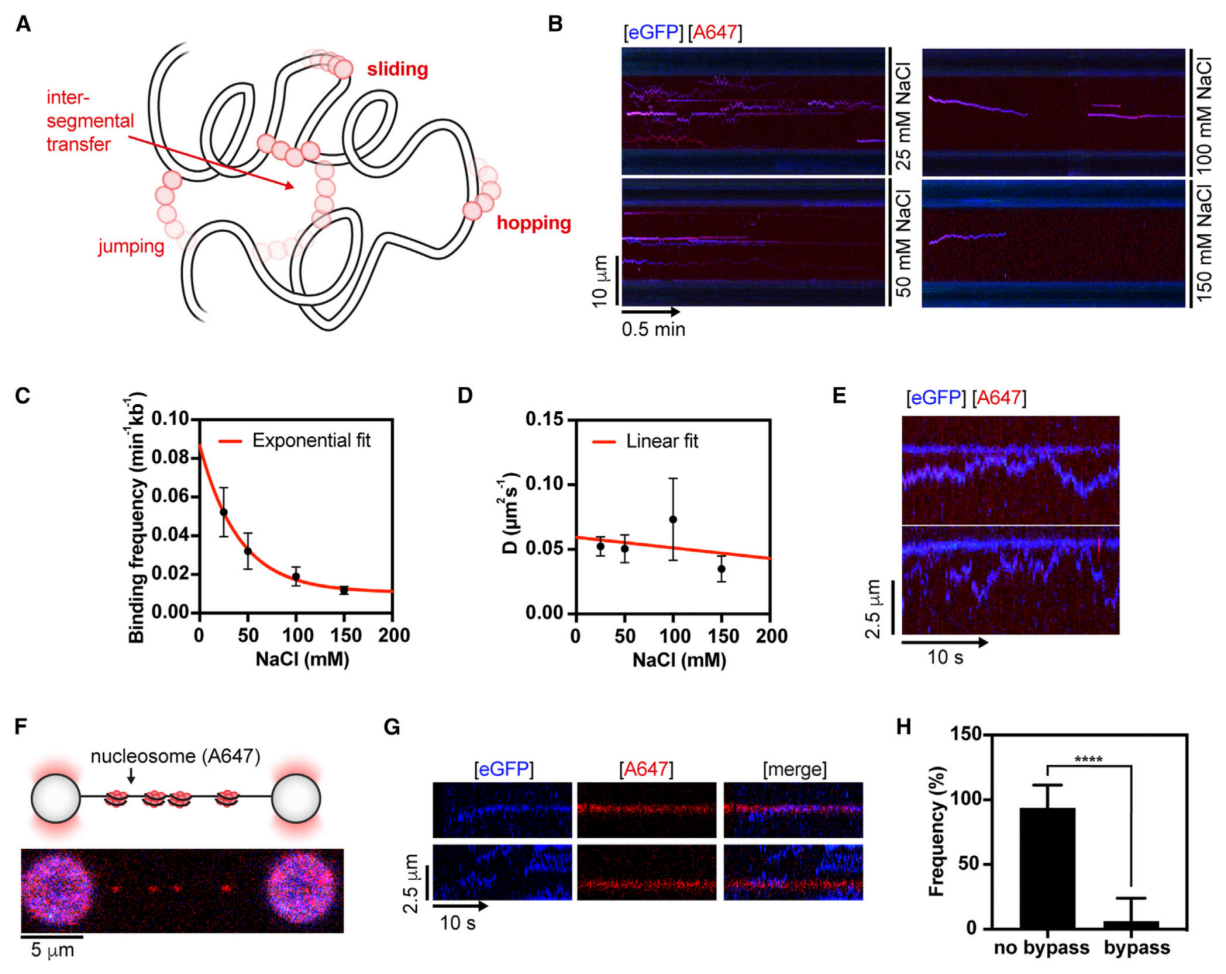
BRCA2 moves by sliding along dsDNA backbone (A) Target search models for DNA-binding proteins. Increasing ionic strength increases the diffusion coefficient during hopping but not sliding. (B) Representative kymographs demonstrating diffusion of BRCA2-RAD51 complex along λ dsDNA at 10 pN at the indicated salt concentrations. 2 nM BRCA2-eGFP (blue), 20 nM RAD51(A647) (red). (C) Binding frequency (n = 9–11) calculated for BRCA2-RAD51 complexes as a function of salt concentration. Error bars represent SEM. (D) Corrected diffusion coefficient (N = 8–24, exclusion of fraction in 0.01 mm^2^s^−1^ bin) calculated for BRCA2-RAD51 complexes as a function of salt concentration. Error bars represent SEM. (E) Representative kymographs demonstrating collision between fast-moving and slow-moving BRCA2-RAD51 complex along λ dsDNA held at 10 pN force. 2 nM BRCA2-eGFP (blue), 20 nM RAD51(A647) (red), 50 mM NaCl, 2 mM MgCl_2_, and 2 mM ATP. (F) Sparsely chromatinized λ DNA substrate (F = 5 pN). Nucleosomes are fluorescently labeled on H4-E63C with Alexa Fluor 647. (G) Representative kymographs showing BRCA2/RAD51 complex collisions with labeled nucleosomes. 2 nM BRCA2-eGFP (blue), 20 nM RAD51 (dark), labeled nucleosomes in red. F = 5 pN. 50 mM NaCl, 2 mM MgCl_2_, and 2 mM ATP. (H) Frequency of collision outcomes for BRCA2-eGFP/RAD51 complexes with labeled nucleosomes on individual chromatinized γ DNA molecules (n = 8). BRCA2 cannot bypass a nucleosome barrier, indicating a tight association with dsDNA during sliding. Error bars represent SD. p values by Student’s t test. n.s., p > 0.05; *p ≤ 0.05; **p ≤ 0.01; ***p ≤ 0.001; ****p ≤ 0.0001. See also Figure S4.

DNA-binding proteins can diffuse along dsDNA by multiple different mechanisms. Among these are sliding and hopping on linear DNA (1D) or jumping and inter-segmental transfer on DNA coiled in 3D space^38–41^ (Figure 5A). To further characterize the mode of BRCA2-RAD51 complex diffusion on dsDNA, we measured the diffusion coefficient in the presence of different NaCl concentrations. Owing to DNA shielding by cations, hopping is expected to increase the diffusion coefficient as a function of salt concentration, whereas sliding does not.^35,42,43^ We probed the diffusion of the BRCA2-RAD51 complex in the presence of 25–150 mM NaCl (Figure 5B). Increasing salt concentrations decreased the overall DNA-binding frequency of BRCA2 (Figure 5C) but did not increase the diffusion coefficient. The diffusion coefficient of BRCA2 instead decreases linearly with salt concentration (by a factor of 0.20 ± 0.04 μ m^2^s^−1^M^−1^, R^2^ = 0.93, Figure S4A). When the data are inspected more closely, the apparent decrease seems to be due to an increase in the proportion of static/confined BRCA2 molecules rather than an overall shift in distribution toward lower diffusion coefficients (Figure S4B). When this was corrected by excluding the slowly diffusing and static fraction (D ≤ 0.01 μ m^2^s^−1^: size of the first bin in Figure 4F) from the analysis, the corrected diffusion coefficient is not significantly salt-dependent (0.09 ± 0.19 μ m^2^s^−1^M^−1^, R^2^ = 0.24, Figure 5D). These results indicate BRCA2 diffuses predominantly via sliding on dsDNA. In support of an intimate association of BRCA2 with dsDNA during sliding, we noted that fast-moving BRCA2 species do not jump over static/slowly moving BRCA2 species (Figure 5E).

Given that dsDNA is chromatinized in cells, we next questioned how a naturally occurring barrier—a nucleosome particle—may affect the sliding of BRCA2. To this end, we labeled histone H4 E63C at C63 with Alexa Fluor 647-maleimide (referred to as H4 E63C-AF647), and salt dialysis was used to assemble histone octamers containing H2A, H2B, H3, and H4 E63C-AF647 into sparsely bound A647-labeled nucleosomes on biotinylated λ DNA, as described previously.^44^ We then tethered sparsely chromatinized λ DNA between two streptavidin-coated beads held at low force ≤5 pN (Figure 5F) and confirmed the presence of DNA-wrapped nucleosome particles by the formation of ~25 and ~50 nm rips^45^ in the force-extension curve of chromatinized λ DNA (Figures S4C and S4D), which roughly correspond to dual- or single-unwrapping events of 147 bp of DNA wrapped around a nucleosome.^46^ As previously reported, unwrapped labeled histones persist on λ DNA even at high forces^47^ (Figure S4C). We then monitored the impact of labeled nucleosome particles on the ability of BRCA2 to slide on dsDNA (Figure 5G). We observed that in >90% of cases, sliding BRCA2 molecules fail to bypass a nucleosome particle (Figure 5H).

These data further support that BRCA2 slides by 1D diffusion on dsDNA and is intimately associated with the dsDNA backbone.

### Differential contribution of BRCA2 DNA-binding domains to dsDNA sliding

**Figure 6:**
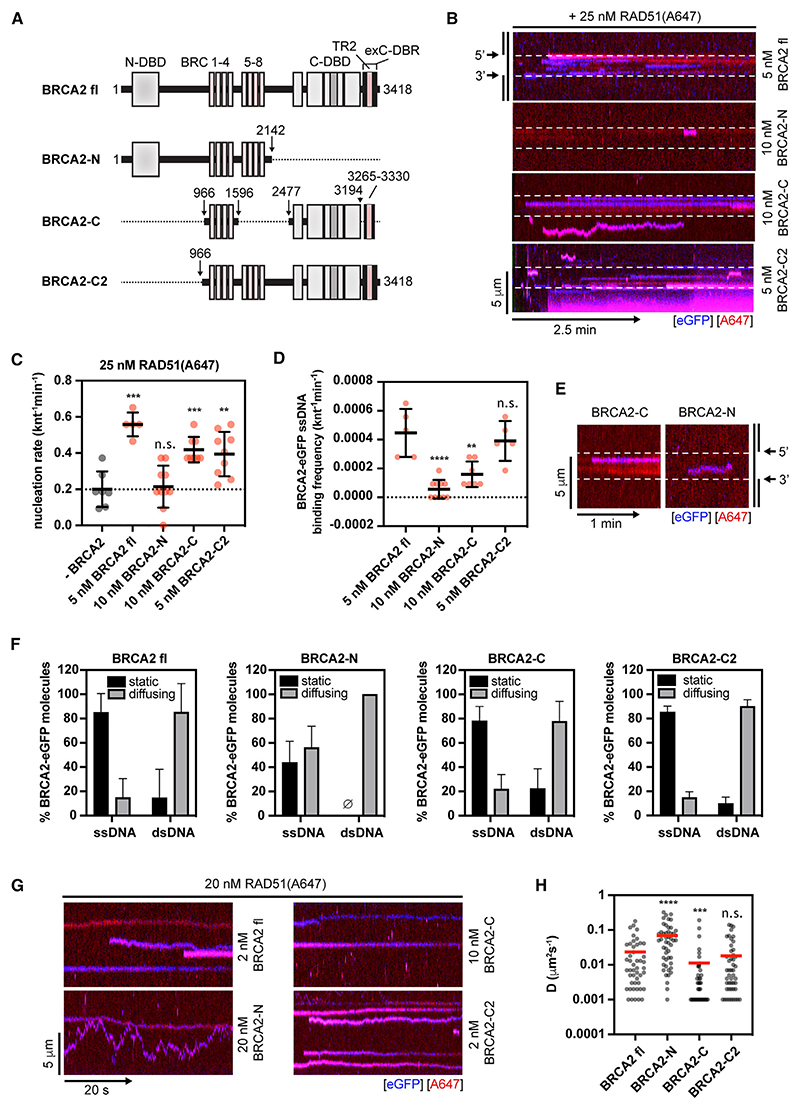
Analysis of BRCA2 mutants (A) A schematic of full-length BRCA2 and deletion mutants. DNA and RAD51 interaction sites are shown. Dotted line represents deleted regions. (B) Representative kymograph showing the binding of 25 nM RAD51(A466) (red) to λ gDNA in the presence/absence of the indicated concentration of full-length (fl) BRCA2 or mutants fused to eGFP (blue) in the presence of 5 nM SYTOX Orange, 100 mM NaCl, 2 mM MgCl_2_, 1 mM CaCl_2_, and 2 mM ATP at 15 pN force. Position of the ssDNA gap is indicated by dashed lines. (C) Quantification of apparent RAD51(A647) nucleation rates in the first 120 s of imaging in the indicated conditions. Error bars represent SD. p values by Student’s t test. (D) ssDNA-binding frequency of BRCA2-eGFP fl and mutants in the first 120 s window at indicated concentrations in the presence of 20 nM RAD51(A647), 100 mM NaCl, 2 mM MgCl_2_, 1 mM CaCl_2_, and 2 mM ATP. Error bars represent SD. p values by Student’s t test. (E) Representative kymograph demonstrating the unstable (mobile) nature of BRCA2-N binding to ssDNA. (F) Fraction of diffusive or static BRCA2-eGFP molecules for different constructs on ss- (n = 8 for BRCA2-N, n = 11 for BRCA2-C, and n = 11 for BRCA2-C2) or dsDNA (n = 2 for BRCA2-N, n = 6 for BRCA2-C, and n = 8 for BRCA2-C2). Error bars represent SEM. Data for BRCA2 fl are the same as in Figure 4B. (G) Representative kymographs showing BRCA2 fl/RAD51, BRCA2-C/RAD51, or BRCA2-N/RAD51 complex diffusion on dsDNA. BRCA2-eGFP concentration is indicated (blue) and 20 nM RAD51 (red). F = 10 pN. 50 mM NaCl, 2 mM MgCl_2_, and 2 mM ATP. (H) Diffusion coefficient (D) calculated from MSD analysis for BRCA2 fl or mutant-RAD51 complexes moving on dsDNA. BRCA2-eGFP concentration is indicated, 20 nM RAD51(A647), 10 pN force, 50 mM NaCl, 2 mM MgCl_2_, and 2 mM ATP. N = 48–60 molecules. Mann-Whitney test. Red line represents mean. (C–I) n.s., p > 0.05; *p ≤ 0.05; **p ≤ 0.01; ***p ≤ 0.001; ****p ≤ 0.0001. See also Figure S5.

BRCA2 is a long polypeptide containing multiple functional modules. To understand which domains of BRCA2 contribute to the dsDNA sliding activity, we generated three deletion mutants (Figures 6A and S5A): (1) a construct containing the N-DBD and BRC repeats 1–8, but lacking the C-terminal DNA-binding domain (C-DBD), C-terminal RAD51-interacting domain (TR2), and exC-DBR—referred to as BRCA2-N; (2) a construct containing BRC repeats 1–4, TR2 (residues 3,265–3,330) and C-DBD, but lacking N-DBD and full context of exC-DBR—referred to as BRCA2-C; (3) a construct containing BRC repeats 1–8, C-terminal DBD, TR2 region as well, and full exC-DBR (residues 3,194–3,418), but lacking N-DBD—referred to as BRCA2-C2. We note that multiple reports have demonstrated that isolated TR2 (residues 3,265–3,330) lacks detectable ssDNA and dsDNA-binding activity even at very high concentrations (up to 4 μM) of the peptide.^6–8,48^

First, we monitored the ability of the deletion mutations to stimulate RAD51 nucleation on ssDNA (Figures 6B and 6C). BRCA2-C2 stimulated RAD51 nucleation to a slightly lower extent than fl BRCA2, BRCA2-C also stimulated RAD51 nucleation but at increased concentrations, whereas BRCA2-N failed to stimulate RAD51 nucleation even at high concentrations (Figure 6C). The ability to nucleate RAD51 correlated well with ssDNA-binding frequencies (Figure 6D) and affinities determined by electrophoretic mobility shift assays (EMSAs) (Figures S5B and S5C). The unstable DNA binding of BRCA2-N is further evident from the increased fraction of mobile molecules even on ssDNA portion of the gDNA substrate (Figures 6E and 6F). When monitoring the sliding of individual BRCA2 mutants (Figures 6G and 6H), BRCA2-C2 displayed similar, albeit slightly slower sliding rates than fl BRCA2, and BRCA2-C sliding rates were significantly slowed compared with fl BRCA2. Lastly, purified isolated exC-DBR-eGFP (residues 3,169–3,418; Figures S5D and S5E) displayed even slower diffusion than the other constructs (Figures S5F and S5G). Interestingly, BRCA2-N displayed faster sliding than fl BRCA2, which is likely due to the unstable nature of its interaction with DNA.

## Discussion

### Mechanism of BRCA2 recruitment to DSBs and BRCA2-mediated RAD51 nucleation

**Figure 7:**
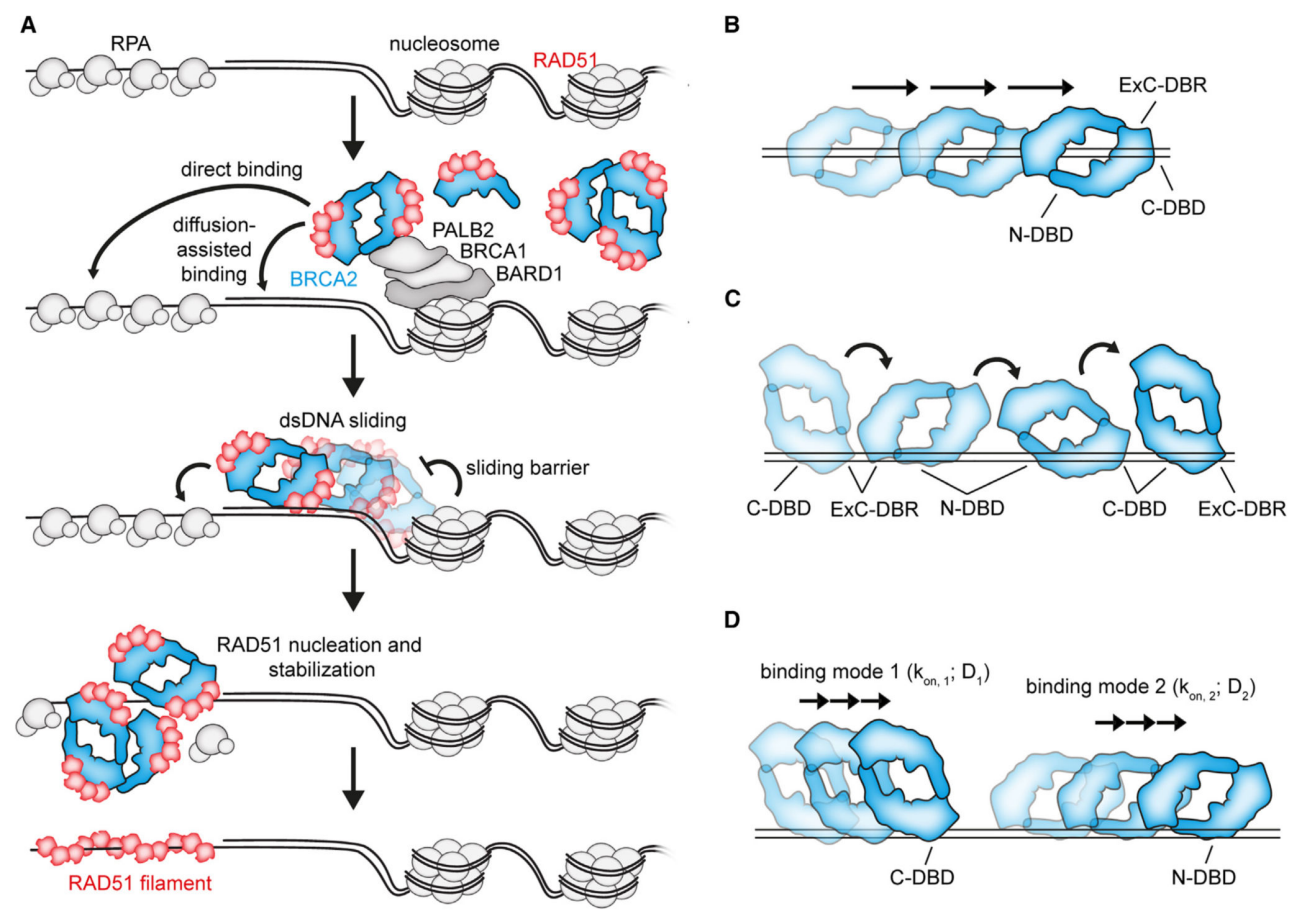
Model for RAD51 nucleation and dsDNA sliding by human BRCA2 (A) BRCA2-RAD51 complex is recruited to the proximity of DSBs. BRCA2 then either nucleates RAD51 directly on RPA-coated ssDNA or slides on the dsDNA arm. During sliding, proximal nucleosomes serve as a diffusion barrier, restricting the sliding of BRCA2 toward resected RPA-coated ssDNA. RAD51 is nucleated and stabilized on ssDNA by BRCA2 and grows into a filament. (B) A hypothetical model of BRCA2 sliding via dsDNA passing through a central channel within the BRCA2 structure. (C) A hypothetical model of BRCA2 sliding via multivalent dsDNA interactions, where dsDNA is shuttled between the different BRCA2 DNA-binding modules. (D) A hypothetical model of BRCA2 sliding on dsDNA via individual DNA-binding modules with different sliding rates and DNA affinities. A mixed mode of movement, where hopping has a contribution, is also possible. (A–D) For simplicity, BRCA2 is shown as a dimer. However, BRCA2 could engage in the proposed sliding mechanisms in monomeric form or as a heterogeneous oligomer.

BRCA2 domains mediating ssDNA and/or dsDNA-binding activity have been previously identified. Although ssDNA binding has been implicated in facilitating the recruitment and nucleation of RAD51 to DNA damage sites, the function and relevance of dsDNA binding by BRCA2 have remained poorly understood. In this work, we used SM approaches to directly observe BRCA2-mediated RAD51 nucleation and stabilization on ssDNA. Unexpectedly, our study has revealed the existence of two modes of nucleation: (1) direct, where BRCA2 nucleates RAD51 on ssDNA and/or ds-ssDNA junctions and (2) diffusion assisted, where mobile BRCA2/RAD51 complexes bind to and slide along dsDNA, delivering RAD51 at the edges of ssDNA gaps. Importantly, BRCA2 sliding is constrained by physiological barriers, such as nucleosomes. Notably, upon DSB formation in cells, H2A lysine 15 ubiquitination on proximal nucleosomes is read by BRCA1-BARD1.^49–51^ PALB2 then bridges BRCA1 and BRCA2 to facilitate the recruitment of BRCA2-RAD51.^52,53^ Based on our observations, we speculate that, in addition to direct RAD51 nucleation on ssDNA, BRCA2 could use dsDNA as a lattice to slide toward resected ssDNA, with nucleosomes proximal to the DNA break restricting unproductive movement away from the DSB (Figure 7A). Another possibility is that chromatin remodelers re-position histones to aid BRCA2 sliding in proximity to the resected DNA break. Consistent with this, previous data have demonstrated extensive chromatin remodeling around DSBs, with SWI/SNF (SWItch/sucrose non-fermentable), RSC (remodeling the structure of chromatin), and INO80 (INOsitol-requiring mutant 80) playing important roles.^54–56^ Although RSC and SWI/SNF are important for DNA end resection,^54^ chromatin remodeling by INO80 has been involved in RAD51 filament formation.^57^ Lastly, human BRCA1 was shown to physically interact with BRG1 subunit of the chromatin remodeler SWI/SNF complex.^58^

Previous single-particle TIRF (total internal reflection fluorescence microscopy) tracking of BRCA2 revealed that BRCA2 diffuses as oligomeric clusters in live cells,^30^ with RAD51 displaying a very similar diffusive behavior. The basis and importance of this behavior for HR were not readily explained. Analysis of diffusion rates in cells revealed three populations of diffusing BRCA2 molecules: free BRCA2 diffusing in the nucleoplasm (D_1_ = 1.15 μm^2^s^−1^) and two ‘transiently bound’ nuclear fractions (D_2_ = 0.05 μm^2^s^−1^ and D_3_ = 0.003 μm^2^s^−1^).^30^ Strikingly, the diffusion coefficients of transiently bound BRCA2 fractions in cells correspond well to those measured for BRCA2-eGFP molecules sliding on dsDNA (~0.04 μm^2^s^−1^; Figure 4F) or binding in a static fashion in our SM experimental settings (<0.01 μm^2^s^−1^; Figure 4F). Hence, we propose that the behavior of BRCA2/RAD51 in cells reflects their diffusion along dsDNA, which is required for the co-complex to reach and engage with the ssDNA prior to RAD51 nucleation. Because dsDNA binding is too labile to allow RAD51 filaments to nucleate on dsDNA, this would minimize the unproductive formation of RAD51-dsDNA filaments.

### BRCA2 contains multiple DNA-binding modules important for RAD51 nucleation, DNA binding, and DNA sliding

BRCA2 contains multiple DNA-binding domains contributing to ss and dsDNA binding. The C-DBD was shown to bind ssDNA with 10–50 nM affinity and dsDNA in the higher nanomolar range.^5^ Within the tower domain of C-DBD, the three-helix bundle (3HB) is present. 3HB has structural similarity to dsDNA-binding domain of bacterial site-specific recombinases,^59^ raising the possibility it could bind dsDNA within some context. The N-DBD bears similarity to the DNA-binding domain of PARP1 and binds ssDNA and dsDNA with similar affinities.^10,60^ Most recently, a third BRCA2 DNA-binding module was described^8^ within the unstructured region at the extreme C terminus of BRCA2 (exC-DBR). exC-DBR was shown to have an auxiliary role in BRCA2 DNA binding, particularly dsDNA. C-DBD and exC-DBR seem to form a functional unit critical for high-affinity DNA binding by BRCA2.^8^ Our data suggest that BRCA2 can bind both ssDNA and dsDNA through its C-DBD, with an intact exC-DBR potentiating DNA binding of BRCA2 in our SM assays. Interestingly, we note that a BRCA2 mutant lacking the N-DBD diffuses slower on dsDNA compared with fl BRCA2. These data suggest that the N-DBD and intact exC-DBR partially contribute to fast movement of BRCA2 on dsDNA. Our data show that N-DBD has a less prominent effect on BRCA2 DNA binding, as an isolated N-DBD-BRC1-8 truncated protein shows low DNA-binding frequency, does not promote strong RAD51 nucleation, and engages with DNA in a highly unstable fashion given its high mobility and diffusion coefficient compared with fl BRCA2. These observations are consistent with single-particle tracking of BRCA2 truncations lacking C-DBD, exC-DBR, or both in murine embryonic stem cells,^61^ where C-DBD or exC-DBR deletion had a modest effect on the overall diffusive behavior of BRCA2, but combined deletion of C-DBD and exC-DBR resulted in a significant reduction of immobile BRCA2 fraction following IR treatment, which is consistent with the labile DNA binding of BRCA2-N in our system.

In agreement with our data, exC-DBR BRCA2 truncation mutant containing two BRC repeats and C-DBD was shown to be a minimal efficient unit capable of modest complementation of BRCA2-deficient cells in HR assays.^62^ Addition of the extreme C terminus, which contains exC-DBR and binds RAD51 increases HR efficiency further.^62^ A truncation containing the N-terminal PALB2-binding site (residues 1–200), two BRC repeats, C-DBD, and exC-DBR leads to a further increase in HR efficiency, highlighting the importance of the PALB2-BRCA2 interaction. Further addition of the N-terminal residues constituting the N-DBD (residues 200–500) resulted in a significant, albeit small, increase in HR efficiency.^62^ Fusing only two BRC repeats, exC-DBR, and the PALB2-binding site (micro-BRCA2) also resulted in a small but detectable complementation. Our SM data provide mechanistic insight into these genetic observations, with individual BRCA2 DNA-binding modules each contributing to ssDNA/dsDNA binding and/or dsDNA sliding, albeit to different extents. Overall, we observed that DNA-binding frequency and sliding speed correlate well with the assays measuring HR efficiency performed using similar BRCA2 thruncations.^62^ Isolated exC-DBR slides the slowest, followed by BRCA2-C, BRCA2-C2, and fl BRCA2 sliding the fastest. However, we note that these are correlative observations, and additional work is required to provide direct evidence for BRCA2 DNA sliding in cells and its importance for HR. Consistent with BRCA2 DNA-binding sites acting as individual modules, targeted deletion of C-DBD^62^ or exC-DBR^63^ was reported to have only a moderate impact on HR efficiency, with mice bearing extreme C-terminal deletion^64^ being viable, contrasting with the early embryonic lethality observed with complete BRCA2 ablation. The survival of mice bearing this BRCA2 deletion could also be explained by compensating parallel DSB repair pathways such as RAD52-dependent single-strand annealing and POLQ (DNA polymerase theta)-dependent microhomology-mediated end joining, both known to be synthetically lethal with BRCA2 loss in cells.^65–67^ Lastly, independently isolated PARP inhibitor-resistant BRCA2 mutants frequently contained intragenic deletion of C-DBD.^68^ This suggests that, in cancers, N-DBD and exC-DBR (or binding partners such as PALB2) can compensate for the loss of the C-DBD and constitute resistance to targeted therapy, supporting the notion that BRCA2 acts as a modular protein.

### The importance of BRCA2 dsDNA binding and sliding in the context of its diverse roles in DNA metabolism

The observed diversity of BRCA2-DNA interaction modes conferred by multiple DBDs may reflect the differential requirements for BRCA2-mediated RAD51 assembly at different endogenous substrates, including resected DSBs, stalled or reversed RFs, or post-replicative ssDNA gaps. BRCA2 binding partners, including PALB2^53^ and DSS1, could also influence its dsDNA binding and sliding properties. PALB2 binds dsDNA and branched DNA structures^69,70^ and synergizes with piccolo variants of BRCA2 in stimulating RAD51 strand exchange.^69^ It is, therefore, possible that the DNA-binding domains of PALB2 cooperate with the dsDNA-binding modules of BRCA2. Similarly, DSS1 was shown to bind BRCA2 within the C-DBD^5^ in proximity to the exC-DBR, which might further influence the dsDNA binding and sliding properties of BRCA2.

*In vitro* nuclease protection assays using RAD51 and BRCA2 peptides suggested that the presence of a RAD51 nucleus in proximity to a ds-ssDNA junction is important for suppressing MRE11-dependent DNA degradation, whereas targeting of RAD51 to ssDNA regions located further from the junction is not.^48^ In a separate study, a mutant of the extreme BRCA2 C terminus that abolishes BRCA2 dsDNA binding within the context of a minimal BRCA2 truncation construct (miniBRCA2) was shown to confer DNA damage sensitivity, decrease RAD51 focus formation, and increase nascent strand degradation in cells.^8^ Lastly, a recent study^71^ has demonstrated that an isolated BRCA2 N terminus binds dsDNA. A single-point mutation that abolishes dsDNA-binding activity of this region resulted in impaired RF protection and compromised ssDNA gap repair.^71^ Collectively, these observations raise a paradox, as earlier studies have demonstrated that BRCA2 strongly disrupts the formation of ‘unproductive’ RAD51 filaments on dsDNA.^3,12,14^ Our direct SM observations explain this as the strikingly labile interaction of BRCA2-RAD51 complex with dsDNA coupled with sliding does not allow for stable RAD51 filamentation on dsDNA but rather delivers RAD51 onto ss-dsDNA junctions where it can protect dsDNA from nuclease-mediated degradation. We also note that diffusion-assisted delivery of BRCA2/RAD51 by dsDNA sliding would allow RAD51 to be delivered to both 3’ and 5’ ds-ssDNA junctions (as we observed). Although delivery to 5’ ds-ssDNA junction due to intrinsic affinity of BRCA2 for this structure would be beneficial in the context of DSB repair, diffusion-assisted delivery to 3’ ds-ssDNA junctions could be critical for protection against MRE11-dependent degradation during DNA replication, consistent with the aforementioned phenotypic observations.

### Potential structural basis for dsDNA sliding activity of BRCA2

Our SM experiments also support previous *in vitro* and cellular data suggesting that BRCA2 is a heterogeneous multimeric protein, with BRCA2 monomers/dimers being the most prevalent species. BRCA2 was shown to multimerize via multiple contacts. Negative stain reconstructions of a BRCA2 dimer suggested N-to-C association, with the C termini being far apart when visualized by antibody labeling.^17^ N-to-N and C-to-C BRCA2 associations were detected by *in vitro* pull-down assays performed with BRCA2 fragments.^19^ Although a high-resolution fl BRCA2 structure is currently unavailable, low-resolution reconstructions display several distinct features, such as the formation of elongated complexes with a central channel formed by two L-shaped BRCA2 monomers.^17,20^ ssDNA was proposed to bind along the axis of the BRCA2 dimer, supported by the C termini localized on the two ends of the dimer.^17^

How BRCA2 slides on dsDNA is not clear. Given that DNA sliding is common to proteins with a clamp-like structure, such as PCNA (proliferating cell nuclear antigen),^72^ it is tempting to speculate that dsDNA may pass through a DNA-binding interface on the central channel of the BRCA2 dimer (Figure 7B). Yet, DNA channel formation within the context of a BRCA2 monomer is also a possibility. Alternatively, the different DNA-binding domains of BRCA2 may cooperate in dsDNA sliding via a hand-over mechanism, as proposed for multivalent DNA binders such as SMC (structural maintenance of chromosomes) proteins^73^ (Figure 7C). Lastly, individual BRCA2 DBDs or DBD pairs may act as independent units, each contributing to 1D diffusion by distinct mechanisms, or may act by steric constraint (encircling or partially encircling the dsDNA) (Figure 7D). Although sliding seems to be the predominant mechanism of BRCA2 1D-diffusion, we cannot exclude a mixed mode of diffusion where hopping is also involved, albeit to a lesser extent. The exact structural basis for BRCA2 sliding on dsDNA remains to be addressed.

In conclusion, we report direct imaging of BRCA2-mediated RAD51 nucleation. We demonstrate that human BRCA2 is an efficient RAD51 nucleation factor that interacts with DNA in two distinct modes: static binding predominantly on ssDNA and an unappreciated ability to bind to and slide on dsDNA. Distinct DNA-binding modules within BRCA2 contribute to ssDNA and dsDNA association as well as dsDNA sliding, with C-DBD and exC-DBR having a more prominent role than N-DBD. Our work thus provides a detailed description of the molecular events underlying RAD51 nucleation by human BRCA2 protein and its modular nature and versatile mode of interaction with DNA, which have relevance for multiple processes maintaining genome stability during DSB repair, RF protection, and ssDNA gap suppression.

### Limitations of the study

The limited spatial resolution of the system (100 nm/pixel) could influence the overall nucleation/binding rates reported. Given our reductionist approach, other recombination mediators and post-translational modifications are not present, limiting the applicability of our findings to reactions occurring *in vivo*. Our BRCA2 preparations are not perfectly homogeneous; further improvements in protein production would benefit future work.

### Star⋆Methods

#### Key Resources Table

**Table:** 

REAGENT or RESOURCE	SOURCE	IDENTIFIER
Antibodies		
anti-RPA70	Abcam	Cat# ab79398; RRID: AB_1603759
anti-BRCA2	Merck	Cat# OP95-100UG; RRID: AB_213443
anti-BRCA2 (aa 188-563)	Abcam	Cat# ab97; RRID: AB_296572
anti-BRCA2 (aa 1651-1821)	Oncogene (now Millipore)	Cat# OP95; RRID: AB_2067762
Anti-GFP	Roche	Cat# 11814460001; RRID: AB_390913
Anti-FLAG M2	Sigma-Aldrich	Cat# F1804-50UG; RRID: AB_262044
anti-RAD51	Abcam	Cat# ab213; RRID: AB_302856
Goat anti-Mouse IgG (H+L) Highly Cross-AdsorbedSecondary Antibody, Alexa Fluor Plus 800	Thermo Fisher	Cat# A32730; RRID: AB_2633279
Goat anti-Rabbit IgG (H+L) Highly Cross-AdsorbedSecondary Antibody, Alexa Fluor Plus 680	Thermo Fisher	Cat# A32734; RRID: AB_2633283
Bacterial and virus strains		
*E. coli* BLR(DE3)pLysS	Novagen	Cat# 69956-3
*E. coli* DH5alpha	NEB	Cat# C2987H
Chemicals, peptides, and recombinant proteins		
Ampicillin, Sodium Salt	Merck	Cat# 171254
Chloramphenicol	Merck	Cat# 220551
IPTG, Dioxane-Free, High Purity	Merck	Cat# 420322
Halt™Protease and Phosphatase Inhibitor Cocktail	Thermo Fisher	Cat# 78440
anti-FLAG M2 resin	Merck	Cat# A2220
Econo-Pac gravity flow column	BioRad	Cat# 7321010
SP Sepharose Big Beads, 1 L	Cytiva	Cat# 17065703
Bio-Rad Protein Assay Dye Reagent Concentrate	BioRad	Cat# 5000006
Insect-XPRESS™Protein-free Insect Cell Medium	Lonza	Cat# BELN12-730Q
Sf-900™II SFM	Thermo Fisher	Cat# 10902088
DENARASE, 500 kU	c-LEcta	Cat# 20804-500k
Superdex 200 Increase GL10/300 column	GE Healthcare	Cat# 28-9909-44
Slide-A-Lyzer MINI dialysis units	Thermo Fisher	Cat# 96570
VivaSpin 500 centrifugal concentrator	Sartorius	Cat# VS0121
MaxiGeBaFlex dialysis tube	Generon	Cat# D045
β-mercaptoethanol	Sigma-Aldrich	Cat# 101458612
guanidine hydrochloride	Sigma-Aldrich	Cat# 50940
TCEP (tris(2-carboxyethyl)phosphine)	Sigma-Aldrich	Cat# 646547
StrepTrap HP column (1 ml)	Cytiva	Cat# 28907546
HiTrap Heparin HP column (5 ml)	Cytiva	Cat# 17040703
HisTrap HP column (1 ml)	Cytiva	Cat# 17524701
HiTrap Q HP column (1ml)	Cytiva	Cat# 17115301
HiTrap SP HP column (1ml)	Cytiva	Cat# 29051324
100 kDa MWCO Amicon centrifugal filter	Millipore	Cat# Z648043-24EA
HiTrap Heparin HP column (1 ml)	Cytiva	Cat# 17040601
HiTrap Q FF column (5 ml)	Cytiva	Cat# 17515601
RESOURCE Q anion exchange chromatography column	Cytiva	Cat# 17117701
Mono Q 5/50 GL	Cytiva	Cat# 17516601
Alexa Fluor 647 C_2_ maleimide	Thermo Fisher	Cat# A20347
Vivaspin centrifugal concentrator (30 kDa MWCO)	Merck	Cat# Z614246-25EA
Zeba Spin Desalting Columns, 7K MWCO, 0.5 mL	Thermo Fisher	Cat# 89882
Streptavidin Mag Sepharose	Cytiva	Cat# 28985799
Sodium hypochlorite	Merck	Cat# XX0637
Sodium thiosulfate	Merck	Cat# 106512
PLURONIC F-127	Merck	Cat# P2443-250G
Albumin from bovine serum	Sigma-Aldrich	Cat# A7030
HiTrap Heparin HP 1 mL column	Merck	Cat# GE17-0407-01
Streptavidin-coated polystyrene particles 0.5% w/v	Spherotech	Cat# SVP-40-5
Ni-NTA agarose resin	Qiagen	Cat# 30210
Lambda DNA	Thermo Fisher	Cat# SD0011
Recombinant *X. laevis* histones	The Histone Source, Protein Expression and Purification Facility, Colorado State University	N/A
RAD51	This study	N/A
RAD51(A647)	This study	N/A
hRPA	This study	N/A
BRCA2	This study	N/A
BRCA2-eGFP	This study	N/A
BRCA2-N	This study	N/A
BRCA2-C	This study	N/A
BRCA2-C2	This study	N/A
T4 Polynucleotide Kinase	NEB	Cat#M0201S
T4 DNA Ligase	NEB	Cat# M0202S
T4 DNA Ligase Reaction Buffer	NEB	Cat# B0202S
SYTOX Orange Nucleic Acid Stain	Thermo Fisher	Cat#S11368
S. p. Cas9 D10A nickase	IDT	Cat# 1081062
EDTA-free cOmplete protease inhibitor cocktail	Roche	Cat# COEDTAF-RO
PhosSTOP phosphatase inhibitor cocktail	Roche	Cat# PHOSS-RO
4x NuPAGE LDS sample buffer	Themo Fisher	Cat# NP0008
Clarity Western ECL	Bio-Rad	Cat# 1705061
Clarity Max Western ECL	Bio-Rad	Cat# 1705062
Critical commercial assays				
QIAquick PCR Purification Kit	Qiagen	Cat# 28104
Q5 Site-Directed Mutagenesis Kit	New England BioLabs	Cat# E0554
Deposited data				
Original images with cropped areas marked	Mendeley	Mendeley: https://doi.org/10.17632/sgw9t49xyg.1
Original code for kymograph/image analysis	Zenodo	Zenodo: https://doi.org/10.5281/zenodo.7857347
Experimental models				
*Spodoptera frugiperda* (Sf9) insect cells	Crick Cell Services	N/A
*Trichoplusia ni* High Five (BTI-TN-5B1-4) insect cells	Thermo Fisher	Cat# B85502
Oligonucleotides				
Oligonucleotides are listed in Table S1	IDT and Sigma Aldrich	Table S1
Recombinant DNA				
pFBDM-BRCA2-N	This study	N/A
pFBDM-BRCA2-C	This study	N/A
pFBDM-BRCA2-C2	This study	N/A
pFB-BRCA2	This study	N/A
pFB-BRCA2-eGFP	This study	N/A
pBIG1a-hRFA1/hRFA2/hRFA3-his_10_	This study	N/A
pET11c-RAD51	Dr. Lumir Krejci	N/A
pET11c-RAD51 C319S	This study	N/A
pFastBac1-hRFA1	This study	N/A
pFastBac1-hRFA2	This study	N/A
pFastBac1-hRFA3-his_10_	This study	N/A
Software and algorithms				
GraphPad Prism 9	Graphpad	https://www.graphpad.com/scientific-software/prism/
Fiji	Open source	https://imagej.net/Fiji
Matlab R2018b (9.5.0)	MathWorks	https://uk.mathworks.com
Lumicks Pylake	Python package from Lumicks	https://lumicks-pylake.readthedocs.io/en/latest/index.html#
Other				
C-trap optical trapping and confocal microscopy setup	Lumicks	N/A

### Resource Availability

#### Lead contact

Further information and requests for resources and reagents should be directed to and will be fulfilled by the lead contact, Simon J. Boulton (simon.boulton@crick.ac.uk).

#### Materials availability

Plasmids, recombinant proteins, DNA substrates and cell lines are available without restriction upon requests, which should be directed to the lead contact, Simon J. Boulton (simon.boulton@crick.ac.uk).

#### Data and code availability

- Kymographs, confocal fluorescence microscopy images, electron-microscopy images, western blots, protein and DNA gels have been deposited at Mendeley: https://doi.org/10.17632/sgw9t49xyg.1 and is publicly available as of the date of publication. DOI is listed in the key resources table. Imaging and single-molecule data reported in this publication will be shared by the lead contact upon request.
- The original code used to process single-molecule data has been deposited at Zenodo: https://doi.org/10.5281/zenodo.7857347 and is publicly available as of the date of publication. DOI is listed in the key resources table.
- Any additional information required to reanalyze the data reported in this paper is available from the lead contact upon request.

### Experimental Model And Study Participant Details

#### Bacterial strains

DH5α *E. coli* strain (genotype: fhuA2 Δ(argF-lacZ)U169 phoA glnV44 Φ80 Δ(lacZ)M15 gyrA96 recA1 relA1 endA1 thi-1 hsdR17) was transformed with protein expression plasmids (key resources table) and grown in Luria Broth at 37°C in the presence of ampicillin (100 mg/l). BLR(DE3)pLysS *E. coli* strain (genotype: F^−^ *ompT hsdS* (r ^−^ m ^−^) *gal dcm* (DE3) D(srl-recA)306::Tn*10* pLysS (Cam^R^, Tet^R^)) was transformed with protein expression plasmids (key resources table) and grown in Luria Broth at 37°C in the presence of ampicillin (100 mg/l) and chloramphenicol (33 μg/l) with constant agitation (180 rpm). Protein expression was induced at OD of 0.6-0.8 with 1 mM isopropyl β -D-1-thiogalactopyranoside (IPTG) at 37 °C for 3–4 h.

#### Insect cell lines

*Spodoptera frugiperda* (Sf9) cells were manipulated according to the instructions accompanying Bac-to-bac system (Life technologies). Sf9 insect cells were grown in Sf-900 III serum free media (Gibco) and seeded at 3x10^6^ cells/ml for expression of pFB-BRCA2 or pFB-BRCA2-eGFP. For protein production, cells were infected with a multiplicity of infection equal to 5 for 66 hours.

Sf9 cells were grown at 27°C with 140 rpm agitation. *Trichoplusia ni* High Five (BTI-TN-5B1-4) insect cells were grown in Insect-XPRESS (Lonza) at 27°C with 140 rpm agitation and manipulated in a fashion similar to *Spodoptera frugiperda* (Sf9) cells.

### Method Details

#### Expression and purification of full-length BRCA2

*Spodoptera frugiperda* (Sf9) codon-optimised full-length BRCA2 containing a C-terminal Flag_3_ tag was assembled into a pFastBac1 vector, yielding pFB-BRCA2. Modified eGFP containing the mutations A207K, and a preceding (alanine)_8_ linker, were inserted before the Flag_3_ tag by Gibson assembly, yielding the construct pFB-BRCA2-eGFP. High Five cells with the baculoviruses. Insect cells were grown in Insect-XPRESS (Lonza) or Sf900 II SFM (Thermo Fisher Scientific).

For expression of protein in insect cells, bacmids, primary and secondary baculoviruses were generated using the standard procedure outlined in the Bac-to-bac system (Life technologies). RT-PCR was used to quantify baculovirus titers. 1 l of log-phase Sf9 insect cells growing in Sf-900 III serum free media (Gibco) were seeded at 3 × 10^6^ cells/ml, and secondary baculoviruses of pFB-BRCA2 or pFB-BRCA2-eGFP were used to infect the cells with a multiplicity of infection equal to 5 for 66 hours (140 rpm, 27°C). All purification was carried out at 4°C. Cells were harvested by centrifugation (1 500 g, 10 minutes), washed with 1X PBSA (137 mM NaCl, 2.7 mM KCl, 10 mM Na_2_HPO_4_, 1.8 mM KH_2_PO_4_) and re-pelleted. The cell pellet was resuspended in 50 ml lysis buffer (25 mM HEPES pH 7.5, 10% glycerol, 500 mM NaCl, 20 mM MgCl_2_, 5 mM ATP, 0.5% Triton-X100, 0.25 mM TCEP and HALT protease/phosphatase inhibitors) and incubated for 30 minutes with overhead rotation. Chromatin and insoluble matter were cleared by centrifugation (Beckmann J-26 centrifuge and JA25.5 rotor, 60 000 g, 45 minutes). The clarified lysate was incubated with 1 ml FLAG M2 agarose beads for 2 hours. Beads were collected by centrifugation (500 g, 5 minutes) and resuspended with 50 ml purification buffer (25 mM HEPES pH 7.5, 10% glycerol, 0.01% Brij-35, 0.25 mM TCEP) containing 1 M NaCl, 5 mM ATP and 10 mM MgCl_2_ for 30 minutes. Beads were collected by centrifugation (500 g, 5 minutes), transferred to an Econo-Pac gravity flow column (BioRad) and washed with 10 CV of purification buffer containing 500 mM NaCl and 0.5 mM EDTA followed by 20 CV of purification buffer containing 200 mM NaCl and 0.5 mM EDTA. Protein was eluted twice with 1 CV of purification buffer containing 200 mM NaCl, 0.5 mM EDTA and 0.5 mg/ml Flag_3_ peptide (1 hour incubations). 100 μl SP Sepharose beads were transferred to an Econo-Pac gravity flow column (BioRad) and equilibrated with purification buffer containing 200 mM NaCl and 0.5 mM EDTA. Protein eluted from the Flag column were flowed through the SP resin twice and washed with 20 CV purification buffer containing 200 mM NaCl and 0.5 mM EDTA. Protein was eluted from the SP Sepharose by elution with 2 CV purification buffer containing 1 M NaCl and 0.5 mM EDTA. The protein was dialysed into 50 mL of the purification buffer containing 200 mM NaCl and 0.5 mM EDTA, aliquoted, frozen in liquid nitrogen and stored at −80°C. Protein concentrations were measured using protein assay dye reagent (BioRad) and molar concentrations calculated as monomer species. NativePAGE analysis of purified BRCA2 were according to manufacturer guidelines (NativePAGE Novex Bis-Tris Gel System) and the gel was stained using the Colloidal Blue Staining Kit (Invitrogen).

#### Expression and purification of BRCA2 truncations

BRCA2-N (residues 1-2142), BRCA2-C (residues 966-1596, 2477-3194, 3265-3330) and BRCA2-C2 (residues 966-3418) were cloned into pFBDM with a C-terminal eGFP and twin strep tag and the gene was inserted into a bacmid for expression in insect cells using the MultiBac system (PMID: 15568020). Baculovirus particles carrying the bacmids were produced in Sf9 cells and protein was expressed by infecting High Five cells with the baculoviruses. Insect cells were grown in Insect-XPRESS (Lonza) or Sf900 II SFM (Thermo Fisher Scientific). Cells were harvested by centrifugation and stored at -80°C until use.

For protein purification, cells were resuspended in buffer A (20 mM Tris, pH 8, 300 mM KCl, 0.5 mM TCEP) supplemented with 2 mM ATP, 5 mM MgCl_2_, DENARASE nuclease (c-LEcta) and cOmplete protease inhibitor cocktail (Roche) and lysed by sonication. The lysate was clarified by centrifugation at 30 400 x g for one hour and subsequent filtration through a 5 μm and 0.2 μm filter before being loaded onto a 5 ml StrepTrap HP column (Cytiva) using a P-1 pump (Cytiva). The column was washed with 10 ml buffer A supplemented with 0.02% DDM, 2 mM ATP and 5 mM MgCl_2_ followed by a wash with 10 ml buffer A containing 1 M KCl. Protein was eluted with buffer A supplemented with 1 mg/ml desthiobiotin and diluted to 225 mM KCl using buffer A containing 150 mM KCl. The eluate was applied to a 1 ml HiTrap Heparin HP column (Cytiva) and BRCA2 truncations were eluted with a gradient of 150 mM to 400 mM KCl. Fractions containing BRCA2 were pooled and concentrated with a 100 kDa MWCO Amicon centrifugal filter (Millipore) and aliquots were flash frozen in liquid nitrogen.

#### Purification of BRCA2-exC-DBR-eGFP

BRCA2-exC-DBR (exon 26-27, residues 3169-3418) was cloned into a pFBDM vector with an N-terminal sumo tag and a C-terminal eGFP and twin strep tag and the gene was inserted into a bacmid for expression in insect cells using the MultiBac system (PMID: 15568020). Baculovirus particles carrying the bacmids were produced in Sf9 cells and protein was expressed by infecting High Five cells with the baculovirus. Insect cells were grown in Insect-XPRESS (Lonza). Cells were harvested by centrifugation and stored at -80 °C until use. For protein purification, cells were resuspended in buffer A (20 mM Tris, pH 8, 300 mM KCl, 0.5 mM TCEP) supplemented with 2 mM ATP, 5 mM MgCl_2_, DENARASE nuclease (c-LEcta) and cOmplete protease inhibitor cocktail (Roche) and lysed by sonication. The lysate was clarified by centrifugation at 75 000 g for 20 minutes and subsequent filtration through a 5 μm and 0.2 μm filter before being loaded onto a 5 ml StrepTrap HP column (Cytiva) using a P-1 pump (Cytiva). The column was washed with 10 ml buffer A supplemented with 0.02% DDM, 2 mM ATP and 5 mM MgCl_2_ and 1 M KCl followed by a second wash with 10 ml buffer A. Protein was eluted with buffer A supplemented with 1 mg/ml desthiobiotin and 10 mM imidazole and applied to a 1 ml HisTrap column (Cytiva). The HisTrap column was washed with buffer A containing 50 mM imidazole and eluted in buffer A containing 100 mM KCl and 300 mM imidazole. 20 ml of 5 mg/μl 3C protease was added and incubated overnight at 4 °C. The protein was then diluted to 75 mM KCl and applied to a 1 ml HiTrap Q HP column (Cytiva) and eluted with a gradient of 50 mM to 1000 mM KCl in buffer A. The protein eluted at around 150 mM KCl. Fractions containing BRCA2-exC-DBR were pooled and diluted to 100 mM KCL and applied to a 1 ml HiTrap SP HP column (Cytiva) and eluted with a gradient of 50 mM to 1000 mM KCl in buffer A. The protein eluted at around 200 mM KCl. Fractions containing BRCA2-exC-DBR were pooled and concentrated.

#### Expression and purification of RPA

Human *RFA1, RFA2* and *RFA3* with N-terminal his_10_ tag in a pFastBac1 vector were assembled into pBIG1a vector and transfected into Sf9 cells. Titer of baculovirus was determined with baculoQUANT. Sf9 cells (500 ml) were grown in Sf-900 III SFM in a 28°C and infected with P2 baculovirus at 2 million cells/ml (MOI ~ 1) and harvested in 60–66 h. The cell pellet was resuspended in lysis buffer (25 mM HEPES pH 8.0, 500 mM NaCl, 10% glycerol, 20 mM imidazole, 0.25 mM TCEP) supplemented with Halt Protease inhibitors. Sonicated lysate was clarified by centrifugation (49 000 g for 25 min at 4 °C) and loaded onto pre-equilibrated Ni-NTA agarose beads followed by incubation at 4 °C for 1–2 h. The beads were washed with 5 column volumes (CV) of lysis buffer and with 5 CV of lysis buffer containing decreasing NaCl concentration (from 400 to 200 mM). The recombinant RPA was eluted with lysis buffer supplemented with 250 mM imidazole. The recombinant RPA was diluted with buffer D (25 mM HEPES pH 8.0, 100 mM NaCl, 10% glycerol, 0.25 mM TCEP and Halt Protease inhibitors) and loaded onto a pre-equilibrated 6 ml Resource Q column. The protein was eluted with linear gradient 100–600 mM NaCl gradient. Peak fractions containing trimeric RPA were pooled, concentrated, and loaded onto Superdex 200 Increase 10/300 column. The protein was eluted with 25 mM HEPES pH 8.0, 200 mM KOAc, 0.5 mM EDTA, 10% glycerol and 0.25 mM TCEP. The fractions containing pure trimeric RPA were pooled, aliquoted, frozen in liquid nitrogen and stored at -80 °C.

#### Expression, purification, and fluorescent labelling of RAD51 C319S for single-molecule imaging experiments

Recombinant human WT RAD51 was purified as described previously with a few modifications.^24,74^ The pET11c-RAD51 expression vector was transformed into E. coli BLR(DE3)pLysS cells and subsequent culture containing ampicillin (100 mg/l) and chloramphenicol (33 μg/l) was grown to an optical density (OD) at 600 nm of 0.7. RAD51 expression was induced with 1 mM isopropyl β-D-1-thi-ogalactopyranoside (IPTG) at 37 °C for 3–4 h. All of the subsequent steps were performed either on ice or at 4 °C. Cells were collected by centrifugation at 5 000 g. Cell pellets were resuspended in cell breakage buffer (50 mM Tris-HCl pH 7.5, 10% sucrose, 0.5 mM EDTA, 1 M KCl, 1 tablet per 50 ml of protease inhibitor cocktail tablets (Roche), 1 mM PMSF, 1 mM DTT and 0.01% NP-40), sonicated and centrifuged at 100 000 g for 1 h. To precipitate RAD51, 0.242 g/ml ammonium sulphate was mixed with clarified supernatant and centrifuged for 20 min at 10 000 g. The pellet was resuspended with buffer K (20 mM K_2_HPO_4_ pH 7.5, 10% glycerol, 0.5 mM EDTA, 1 mM DTT, 0.01% NP-40) and loaded onto the Q Sepharose Fast flow column (Cytiva), pre-equilibrated with K buffer-low (K buffer supplemented with 175 mM KCl). The column was washed extensively with K buffer-low and protein was subsequently eluted with a KCl gradient using K buffer-high (K buffer supplemented with 0.6 M KCl). The eluted fractions containing RAD51 were pooled and diluted with 6 volumes of dilution buffer (25 mM Tris HCl pH 7.5, 0.5 mM EDTA, 1 mM DTT, 0.01% NP-40). The diluted sample was loaded onto the HiTrap Heparin HP affinity column (Cytiva), which was pre-equilibrated with buffer H without glycerol (25 mM Tris HCl pH 7.5, 0.5 mM EDTA, 1 mM DTT, 0.01% NP-40, 150 mM KCl) and washed with buffer H containing 10% glycerol. Protein was eluted in buffer H with a KCl gradient (0.1 M to 1 M KCl) and the fractions containing RAD51 were pooled and dialysed in buffer H without glycerol. The dialysed sample was loaded onto the Mono Q 5/50 GL column (Cytiva), equilibrated with buffer Q (25 mM Tris HCl pH 7.5, 0.5 mM EDTA, 1 mM DTT, 0.01% NP-40, 100 mM KCl, 10% glycerol) and the column was further washed with buffer Q containing 50 mM KCl but lacking glycerol. RAD51 was eluted from the Mono Q column with a KCl gradient (0.05 M to 1 M) in buffer Q lacking glycerol. The eluted fractions containing RAD51 were pooled and further concentrated with the Vivaspin Centrifugal Concentrator (30 kDa molecular weight cut-off (MWCO)). Glycerol was added to the concentrated sample to a final concentration of 10%. Finally, the samples were aliquoted, frozen in liquid nitrogen and stored at -80 °C.

RAD51 C319S variant was expressed and purified as described earlier for WT RAD51. After purification, the protein was fluorescently labelled with Alexa Fluor 647 C_2_ maleimide (Thermo Fisher Scientific, A20347) according to previously described protocol.^24,25,75^ Briefly, DTT-free 27 μM RAD51 was buffer-exchanged into labelling buffer (50 mM HEPE pH 7.0, 300 mM KCl, 1 mM EDTA, 10% glycerol, degassed for 30 min prior to use) and mixed with 5-fold molar excess of Alexa Fluor 647 C_2_ maleimide (Thermo Fisher Scientific, A20347). Reaction was gently mixed for 2 h at 4 °C. Labelling was terminated by the addition of DTT to 10 mM concentration and further 30 min incubation at 4 °C. Labelled protein was purified away from the free dye using the Zeba column gel filtration system (0.5 ml resin, 7 kDa MWCO) and buffer exchanged into storage buffer (25 mM Tris HCl pH 7.5, 0.5 mM EDTA, 1 mM DTT, 0.01% NP-40, 150 mM KCl, 10% glycerol). The protein concentration was estimated by Coomassie staining and dye concentration was measured spectrophotometrically. The presence of minimum free dye concentration was assessed using SDS–PAGE on labelled proteins. The protein to dye concentration ratio was consistently 0.9–1.0 consistent with previous reports.^24,25,75^

#### Western blotting of BRCA2-eGFP

BRCA2-eGFP was mixed with SDS running buffer (62.5 mM Tris-HCl pH 6.8, 2% [v/v] SDS, 10% [v/v] glycerol, 0.01% [w/v] bromophenol blue, 240 mM B-mercaptoethanol), boiled for 5 minutes at 90 °C and separated on Novex NuPAGE 4-12% BisTris gel run with MOPS in an XCell SureLock Mini-Cell tank at 130 V for 90 minutes. Samples were run alongside a Precision Plus Prestained Protein ladder. Proteins were transferred to a 0.2 μm nitrocellulose membrane in Western blot transfer buffer (25 mM Tris pH 7.5, 190 mM glycine, 0.1% [v/v] SDS, 20% (v/v) methanol) at 350 mA for 90 minutes at 4 °C. Membranes were blocked with Western blocking buffer (TBST [50 mM Tris pH 7.5, 150 mM NaCl, 0.05% Tween 20], 5% (v/v) skimmed milk powder) for 1 hour at room temperature, followed by the addition of primary antibodies in Western blocking buffer to the membrane (overnight, 4 °C). Primary antibodies used to probe BRCA2-eGFP include: anti-BRCA2 (aa 188-563) (Abcam ab97 [3E6], 1:500, mouse monoclonal, batch #GR3796-3), anti-BRCA2 (aa 1651-1821) (Oncogene OP95 [Ab-1], 1:500, mouse monoclonal, batch #D22468-1), anti-GFP (Roche 11814460001 [clones 7.1 and 13.1], 1:500, mouse monoclonal) and anti-FLAG (Sigma F1804 [M2 affinity], 1:1000, mouse monoclonal, batch #SLCF4933). Membranes were washed with TBST (3 x 10 minutes, 25 °C), and incubated with Goat anti-Mouse IgG Highly Cross-Adsorbed Secondary Antibody Alexa Fluor Plus 800 (Invitrogen A32730, 1:1000) in Western blocking buffer (1 hour, 25 °C). Membranes were washed with TBST (3 x 10 minutes, 25 °C), and then imaged on an Odyssey DLx.

#### Electrophoretic mobility shift assay

Full length or truncated BRCA2 at the indicated concentrations was incubated with 4 nM Cy5 labelled 100-mer ssDNA or dsDNA (sequence: 5’-CCAAGAAGCTGTTCAGAATCAGAATGAGCCGCAACTTCGGGATGAAAATGCTCACAATGACAAA-TCTGTCCACGG AGTGCTTAATCCAACTTACCAAGCT) or 3’ tailed DNA (made by annealing two oligos: CTGCTTTATCAAGATAATTTTTCGACT CATCAGAAATATCCGTTTCCTATATTTATTCCTATTA-TGTTTTATTCATTTACTTATTCTTTATGTTCATTTTTTATATCCTTTACTTTATT TTCTCTGTTTATTCATTTACTTATTTTGTATTATCCTTATCTTATTTA and CGGATATTTCTGATGAGTCGAAAAATTATCTTGA-TAAAG CAG) in 20 ml buffer B (20 mM HEPES, pH 7.5, 1 mM MgCl_2_, 1 mM TCEP, 100 mM KCl) for 5 minutes at 24 °C. Protein-DNA complexes were resolved by native gel electrophoresis using 3-12% polyacrylamide gels (Thermo Fisher Scientific) in TAE buffer run at 75 V for 75 minutes. The Cy5 label was visualised using a ChemiDoc MP imaging system (Bio-Rad).

#### Ensemble ssDNA capture assay

RPA and unlabelled RAD51 were used in the magnetic ssDNA pulldown assay. Following standard PCR procedures, a 2023 bp linear dsDNA fragment was amplified using biot-SwaI-2023bp-f (5’-biot-AACAAAATATTAACGCTTACAATTTAAATGGTGGCACTTTTCG-3’) and 2023bp-r (5’-TCAACGTGCAATCAAGTTAATGAATCGG-3’), and gel purified (Table S1). To prepare the ssDNA substrate, the biotinylated 2023 bp dsDNA was bound to Streptavidin Magnetic Sepharose beads (Cytiva), washed with twice with 500 μl 100 mM NaOH to remove the non-biotinylated strand of dsDNA and neutralised with 100 mM HEPES pH 7.5. 1 mM of biot-SwaI-rc (5’ CGAAAAGTGCCACCATTTAAATTGTAAGCGTTAATATTTTGTT-3’) was added to the ssDNA-beads and annealed to the 5’ end of ssDNA to create a short dsDNA SwaI restriction site (rs).

Reactions were performed at room temperature. The SwaI^rs^-ssDNA (300 nM, nt concentration) beads were equilibrated in reaction buffer (25 mM HEPES pH 7.5, 100 mM NaCl, 2.5 mM MgCl_2_, 1 μM ATP, 0.25 mM TCEP, 0.005% Brij), to which RPA (200 nM) and BSA (0.2 mg/ml) were added and incubated for 30 minutes. 100 nM RAD51, with and without 10 nM BRCA2, were then added to the beads (final volume = 100μl) and the reaction was run for 30 minutes with overhead rotation. A control, containing beads only (without ssDNA), was also included to monitor non-specific binding. Beads were washed twice with 500 μl reaction buffer, followed by elution with 30 ml reaction buffer containing 3 U SwaI for 10 minutes with overhead rotation. Eluates were separated and removed from the magnetic beads, mixed with SDS loading buffer and analysed by SDS-PAGE. Gels were transferred to nitrocellulose membranes and blocked with 5% milk in TBST. The following antibodies were applied to cut membranes overnight at 4°C: BRCA2 (mouse, OP95, Merck, 1:500), RAD51 (mouse, ab213, Abcam, 1:1000) and RPA70 (rabbit, ab79398, Abcam, 1:1000). Membranes were washed with TBST (3 x 10 minutes) and incubated with a combined mixture of anti-rabbit Alexa Fluor 680 (A32734, 1:40,000) and anti-mouse Alexa Fluor Plus 800 (A32730, 1:40,000) for 1 hour at room temperature. Membranes were washed with TBST (3 x 10 minutes) and imaged on an Odyssey CLx Infrared Imaging System (Licor).

#### Negative stain electron microscopy

Carbon coated 400 mesh grids (EM Resolutions) were glow-discharged for 30 s at 45 mA. 4 μl of BRCA2 (90 ng/μl) was applied to the grids and incubated for 2 minutes. The grids were negative stained with 2% uranyl acetate, by transferring the grid between four separate 40 μl droplets for a total of 60 seconds. Excess stain was blotted from the grid, and the grid was left to air dry. Samples were loaded onto FEI Tecnai LaB_6_ G^2^ Spirit Transmission Electron Microscope operating at 120 kV with a 4K Gatan OneView camera. Micrographs were collected at a nominal magnification of 30 000 X, equivalent to 3.98 Å/px.

#### DNA substrate preparation for optical trapping and single molecule imaging

Biotinylated λ DNA was prepared as described previously.^16^ Briefly, λ phage DNA was biotinylated at the 3’ and 5’ ends of the same DNA strand. First, the 5’ end of λ DNA and oligonucleotides 1 (5’-GGGCGGCGACCTGGACAA-3’) and 2 (5’-AGGTCGCCGCC CTTTTTTTT(BT)TT(BT)TT(BT)-3’) were phosphorylated for 30 min at 37 °C in a reaction containing 14 nM λ DNA or 10 mM of the oligo-nucleotide and 250 U/ml of T4 PNK in 1 × T4 ligase buffer (NEB). T4 PNK was deactivated for 20 min at 65 °C. Next, oligo-nucleotides 1, 3 (5’-T(BT)TT(BT)TT(BT)TTTTTTTAGAGTACTGTACGATCTAGCATCAATCTTGTCC-3’) and 2 were annealed to the overhangs of λ DNA in a 10:1 oligonucleotide:DNA ratio (total volume 500 μL) by heating the reaction to 80 °C and cooling slowly to room temperature. The ligation reaction then was initiated by adding T4 DNA ligase (20 U/ml) and carried out for 2 h at room temperature. Finally, the DNA was purified by PCR purification kit (Qiagen).

To generate gapped γ DNA precursor, biotinylated hairpin oligonucleotides (5’-AGGTCGCCGCCCGGAGTTGAACG(BT)(BT)T(BT) T(BT)ACGTTCAACTCC-3’ and 5’-GGGCGGCGA CCTCAAGTTGGACAA(BT)T(BT)T(BT)(BT)TGTCCAACTTG-3’) were annealed to γ-dsDNA ends and ligated (Table S1). *Streptococcus pyogenes* Cas9 D10A nickase (IDT) was used to generate two DNA nicks 5.374 bp apart using guide RNAs (tracrRNA: 5’-GGACAGCAUAGCAAGUUAAAAUAAGGCUAGUCCGUUAUCAACUUGAAAAAGU GGCACCGAGUCGGUGCUUUUU-3’; crRNA λ4: 5’-CAGATATAGCCTGGTGGTTCGUUUUAGGAGCUAUGCUGUUUUG-3’; crRNA γ5: 5’-GGCAAUGCCGAUGGCGAUAGGUUUUAGGAGCUAUGCUGUUUUG-3’) described previously (Table S1).^27^ Reaction was terminated and λ gDNA precursor was diluted in filtered PBS directly before use.

λ DNA sparsely-coated with labelled nucleosomes was prepared as described previously.^44^ Briefly, purified, recombinant *Xenopus* histones were purchased from The Histone Source, Protein Expression and Purification Facility, Colorado State University, and their correct molecular mass was verified by mass spectrometry (Proteomics Science Technology Platform, Francis Crick Institute). Histone H4-E63C was labelled with Alexa Fluor 647 C_2_ maleimide (Thermo Fisher Scientific, A20347). First, 150 μM histones were reduced and denatured in 20 μM Tris-HCl (pH 7.5), 10 mM TCEP (Sigma-Aldrich, 646547), and 7 M guanidine hydrochloride (Sigma-Aldrich, 50940) for 30 min at 25 °C. 10 mg/ml Alexa Fluor 647 C_2_ maleimide was mixed dropwise with denatured histones (1:5 v/v ratio). Reaction was carried out for 3 hours at 25 °C. β-mercaptoethanol (Sigma-Aldrich, 101458612) was added in 100-fold molar excess to the dye to quench the reaction. 150 μM H2A, H2B and H3 were individually reduced and denatured in 20 mM Tris-HCl (pH 7.5), 10 mM β-mercaptoethanol, and 7 M guanidine hydrochloride for 3 hours at 25 °C. Denatured histones H2A, H2B, H3, and H4-E63C-Alexa Fluor 647 were mixed at equimolar ratios and adjusted to a total protein concentration of 1 mg/ml with unfolding buffer (20 mM tris-HCl (pH 7.5), 10 mM β-mercaptoethanol, and 7 M guanidine hydrochloride). Denatured histones were loaded into a MaxiGeBaFlex dialysis tube (Generon, D045) and dialyzed at 4°C against 2 l of 10 mM tris-HCl (pH 7.5), 1 mM EDTA, 5 mM β-mercaptoethanol, and 2 M NaCl. Refolding buffer was changed four times. Refolded histones were concentrated via VivaSpin 500 centrifugal concentrator (Sartorius, VS0121) and resolved using Superdex 200 Increase GL10/300 column (GE Healthcare, 28-9909-44) in 10 mM Tris-HCl (pH 7.5), 1 mM EDTA, 5 mM β-mercaptoethanol, and 2 M NaCl at 4°C. Fractions containing stoichiometric octamer pooled, concentrated and flash-frozen in liquid nitrogen. Nucleosome reconstitution was performed by a NaCl gradient dialysis method. For each reconstitution reaction, 1 μg of γ DNA was mixed with a desired molar excess of histone octamer (from 0 to 300 for γ DNA) in 10 mM Tris-HCl (pH 7.5), 1 mM EDTA, and 2 M NaCl at 100 μl final volume, and incubated on ice for 30 min. Reaction was moved into Slide-A-Lyzer MINI dialysis units (Thermo Fisher Scientific, 96570) and dialyzed overnight against 1 l of 10 mM Tris-HCl (pH 7.5), 1 mM EDTA, and 1 M NaCl, then again for 8 h against 1 l of 10 mM Tris-HCl (pH 7.5), 1 mM EDTA, and 0.75 M NaCl, and lastly overnight against 1 liter of 10 mM Tris-HCl (pH 7.5), 1 mM EDTA, and 20 mM NaCl. Chromatinized γ DNA was recovered and stored at 4 °C for 6 months.

#### Single-molecule imaging using dual optical trapping system

Experiments were performed using commercially available C-trap (LUMICKS) setup. Protein channels of the microfluidics chip were first passivated with BSA (0.1% w/v in PBS) and pluronics F128 (0.5% w/v in PBS), minimum 500 ml of both flowed through prior to use. Biotinylated λ DNA or λ gDNA precursor was captured between 4.82 mm SPHERO Streptavidin Coated polystyrene beads at 0.31 pN/nm trap stiffness at 0.005% w/v bead concentration using the laminar flow cell. The presence of ssDNA gap was verified by comparison to built-in worm-like chain model.

For imaging of RAD51 nucleation by BRCA2 fl, gDNA was held at 15 pN force. Beads and DNA were kept in PBS during the experiment. DNA was melted in PBS and visualized in 1xNTMC buffer (50 mM Tris-HCl pH 7.5, 100 mM NaCl, 2 mM MgCl_2_, 1 mM CaCl_2_) supplemented with 0.2 mg/ml BSA, 2 mM ATP and low concentration (1-5 nM) of Sytox Orange, where individual Sytox binding events are distinguishable. gDNA was then moved to channel containing BRCA2-eGFP and RAD51(A647) in indicated concentrations in 1xNTMC buffer (50 mM Tris-HCl pH 7.5, 100 mM NaCl, 2 mM MgCl_2_, 1 mM CaCl_2_) supplemented with 0.2 mg/ml BSA, 2 mM ATP and low concentration (1-5 nM) of Sytox Orange to monitor RAD51 nucleation. In later experiments, 5 nM Sytox Orange was used only in the buffer channel to visualize the ssDNA gap position and left out in channel containing proteins.

For imaging of BRCA2-RAD51 complex movement on dsDNA, λ DNA was held at 10 pN force. Beads and DNA were kept in PBS during the experiment. The presence of a single λ DNA molecules was confirmed in 1xNTM buffer (50 mM Tris-HCl pH 7.5, 50 mM NaCl – or otherwise indicated salt concentration, 2 mM MgCl_2_) supplemented with 0.2 mg/ml BSA, 2 mM ATP. λ DNA was then moved to channel containing BRCA2-eGFP and RAD51(A647) in indicated concentrations in 1xNTM buffer (50 mM Tris-HCl pH 7.5, 50 mM NaCl - or otherwise indicated salt concentration, 2 mM MgCl_2_, 1 mM CaCl_2_) supplemented with 0.2 mg/ml BSA, 2 mM ATP.

For imaging of nucleosome bypass, chromatinized λ DNA was captured and manipulated at low forces (<5 pN). Beads and DNA were kept in PBS during the experiment. After tethering Chromatinized λ DNA was held at 5 pN force in 1xNTM buffer (50 mM Tris-HCl pH 7.5, 50 mM NaCl, 2 mM MgCl_2_) supplemented with 0.2 mg/ml BSA, 2 mM ATP and nucleosomes were visualized by confocal scanning to confirm presence of chromatin. Chromatinized λ DNA was then moved to channel containing BRCA2-eGFP and RAD51 in 1xNTM buffer (50 mM Tris-HCl pH 7.5, 50 mM NaCl, 2 mM MgCl_2_) supplemented with 0.2 mg/ml BSA, 2 mM ATP and held at 5 pN force during the experiment.

For confocal imaging, three excitation wavelengths were used, 488 nm for eGFP and AF488, 532 nm for mStrawberry and AF555 and 638 nm for AF647, with emission detected in three channels with blue filter 512/25 nm, green filter 585/75 nm and red filter 640 LP. Imaging conditions for the initial fl BRCA2 RAD51 nucleation analysis: 6.3 μW blue laser power (7.5 %), 0.9 μW green laser power (7.5 %), 5.5 μW red laser power (5.0 %), 0.1 ms/pixel dwell-time, 100 nm pixel size, 1 s inter-frame wait time. For imaging of diffusing BRCA2-eGFP/RAD51(A647) on dsDNA: 6.5 μW blue laser power (20 %), 12.6 μW green laser power (30 %), 3.0 μW red laser power (5.0 %), 0.1 ms/pixel dwell-time, 100 nm pixel size, 250 ms inter-frame wait time. For imaging of diffusion on chromatinized DNA and diffusion of BRCA2 mutants and fl: 6.5 μW blue laser power (20 %), 3.0 μW red laser power (5.0 %), 0.1 ms/pixel dwell-time, 100 nm pixel size, 250 ms inter-frame wait time. Representative images in figures are displayed as composites.

#### Quantification And Statistical Analysis

Real-time force, distance and fluorescence data were exported from Bluelake HDF5 files and analysed using custom scripts in the Pylake Python package. The worm-like chain model for λ dsDNA was used as a reference for force–extension curve comparison. Binding frequencies and dwell-times were estimated in Fiji. Dwell-time frequency distribution was analysed in GraphPad Prism 9. RAD51 nucleation rates were calculated from number of RAD51 nuclei appearing in the first 60 s of imaging (BRCA2-eGFP fl, ±RPA) or first 120 s (BRCA2 fl comparison with BRCA2-eGFP truncation mutants). Analysis in early timepoints is critical to obtain accurate values due to high RAD51 density on ssDNA in later timepoints. For the position analysis, a per-pixel fraction of total integrated eGFP and AF647 fluorescence intensity in the area of the background-level Sytox Orange signal, corresponding to 5.374 knt long ssDNA gap, was extracted for the section of kymograph starting with first pixel of the fist BRCA2-eGFP/RAD51(A647) binding event and ending with last pixel of the last BRCA2-eGFP/RAD51(A647) binding event recorded. To obtain Tau (τ) values characterizing dwell-time distribution of diffusing BRCA2 molecules at different salt concentrations, BRCA2-eEGFP dwell-times from 31–75 molecules were binned into 7 second binds and resulting histogram was fitted with single exponential function. Mann–Whitney or Student t test were used to assess statistical significance of the data where appropriate with p values indicated in figure legends. Number of molecules, N (either individual DNA molecules or individual labelled protein complexes), is indicated in the figure legend. When estimating a fraction of events occurring during imaging of a molecule, both number of molecules recorded (N) and total number of events for each molecule (n) are indicated in the figure legend.

For analysis of chromatinized λ DNA, force-extension curves were exported from Bluelake HDF5 files and analysed using custom scripts in the Pylake Python package. Force-distance traces were fitted with extensible worm-like chain model (eWLC) as follows:

$$L_{DNA}=L_{C}\left(1-\frac{1}{2}\sqrt{\frac{k_{B}T}{F\times L_{p}}}+\frac{F}{S}\right)$$

Where L_c_ is the calculated contour length of dsDNA (0.34 nm/bp), L_p_ is the persistence length of dsDNA (100 nm), S is the stretching modulus of dsDNA (1000 pN), k_B_ is Boltzmann’s constant, T is absolute temperature (297 K). Best-fit L_c_ for individual parts of the force-distance curve were then compared with the L_c_ difference used to estimate length of dsDNA released from individual chromatin rupture events.

For the mean square displacement (MSD) analysis, we used a custom-made single-particle tracking algorithm in Python,^35^ which was incorporated within each Jupiter Notebook workspace. The kymograph imported from an.h5 file was cropped to eliminate the oversaturated parts of the image (i.e., the bead contours). The sub-pixel position of the fluorescent particle in each frame of the kymograph was calculated by fitting the signal intensity of a three-frame moving window with a 1D Gaussian function (line-time = 0.250 s, 100 nm px^−1^). For the obtained parts of trajectories that were not overlapping with each other, the MSD was calculated using the following equation:

$$MSD\left(n,N\right)=\sum_{i=1}^{N-n}\frac{{\left(X_{I+N}-X_{i}\right)}^{2}}{N-n}=D_{t+b}$$

where *N* is the total number of timeframes in the kymograph, *n* is the number of frames within a moving window (t) from which the square displacement was calculated (ranging from 1 to *N*-1). *X*_*i*_ is the molecule position along the DNA at time *i*, and b is the offset which is a measure for the accuracy. To evaluate whether a trajectory represents random walk or directed motion, the MSD dependency was fitted with a linear function or power law. A particle exhibits free or constrained diffusion with rate *D* when the MSD scales with an exponent α ≤ 1. When α > 1, the process is characterized as super-diffusive motion (for example, unidirectional walk). In the case of free diffusion, a linear fit was used to calculate diffusion coefficient, D. The fitting window was limited to 0.25 < τ < 2 s, to exclude errors emerging from stochastic variations and the confinement of the diffusion due to the limited length of the λ DNA. To estimate the average diffusion speed of the protein, the total route of the labelled protein molecule (a sum of frame-to-frame displacements) was divided by the total trajectory time. Every trajectory was smoothed using the Savitzky–Golay filter (smoothing factor = 51) to eliminate tracking inaccuracies and the molecule’s thermal fluctuations.

## Supplementary Material

Supplemental information

## Highlights

- Single-molecule imaging of full-length human BRCA2 reveals RAD51 nucleation mechanism
- BRCA2 can nucleate and stabilize RAD51 on ssDNA directly
- BRCA2 can slide along dsDNA backbone and deliver RAD51 to ds-ssDNA junctions
- BRCA2 DNA-binding modules differentially contribute to sliding and RAD51 nucleation
