# Supplementary material for: Visualization of direct and diffusion-assisted RAD51 nucleation by full-length human BRCA2 protein
Source: Mol Cell. Author manuscript; Available in PMC 2024 Feb 15. (PMC7615647; doi:10.1016/j.molcel.2023.06.031)
Supplement: Supplemental information [file EMS193943-supplement-Supplemental_information.pdf]

Molecular Cell, Volume *83*

Supplemental information

Visualization of direct and diffusion-assisted RAD51 nucleation by full-length human BRCA2 protein

Ondrej Belan, Luke Greenhough, Lucas Kuhlen, Roopesh Anand, Artur Kaczmarczyk, Dominika T. Gruszka, Hasan Yardimci, Xiaodong Zhang, David S. Rueda, Stephen C. West, and Simon J. Boulton

Supplementary Figures

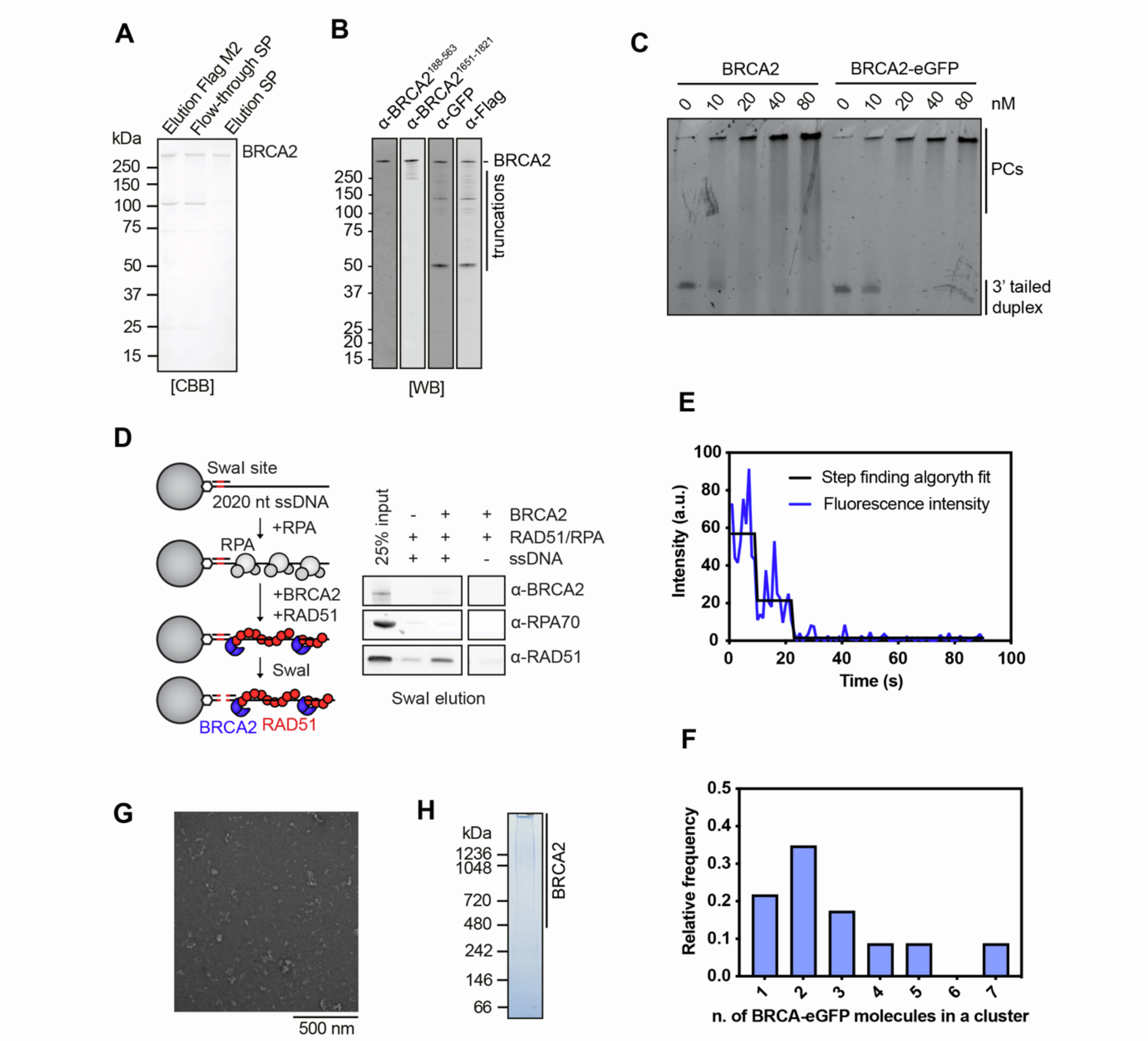

Figure S1: BRCA2 characterization. Related to Fig. 1. (A) Purification of full-length human BRCA2. **(B)** Western blot of purified BRCA2-eGFP performed using antibodies targeting indicated epitopes (N-terminal and central region of BRCA2; eGFP; FLAG). **(C)** Electrophoretic mobility shift assay (EMSA) performed with 167mer 3' tailed DNA and BRCA2 or BRCA2-eGFP. PCs - protein-DNA complexes. **(D)** DNA capture assay. Biotinylated RPA-coated 2020 nt-long ssDNA immobilized on streptavidin-coated beads was incubated with RAD51 and BRCA2, washed and proteins were eluted via SwaI cleavage of the DNA. Samples were analysed by SDS-PAGE. Quantitative analysis was performed following western-blotting and fluorescent detection using Licor system. **(E)** Examples of a fluorescence intensity trace of the BRCA2-eGFP cluster during continuous photobleaching. Black line represents stepping fit of the trace. **(F)** Histogram of BRCA2 cluster size in the presence of RAD51 (N = 23 molecules). **(G)** Electron micrograph of recombinant BRCA2 negatively stained with uranyl acetate. The sample is polydisperse owing to the range of oligomeric states observed. **(H)** 3-12% Native PAGE of recombinant BRCA2, stained with Coomassie.

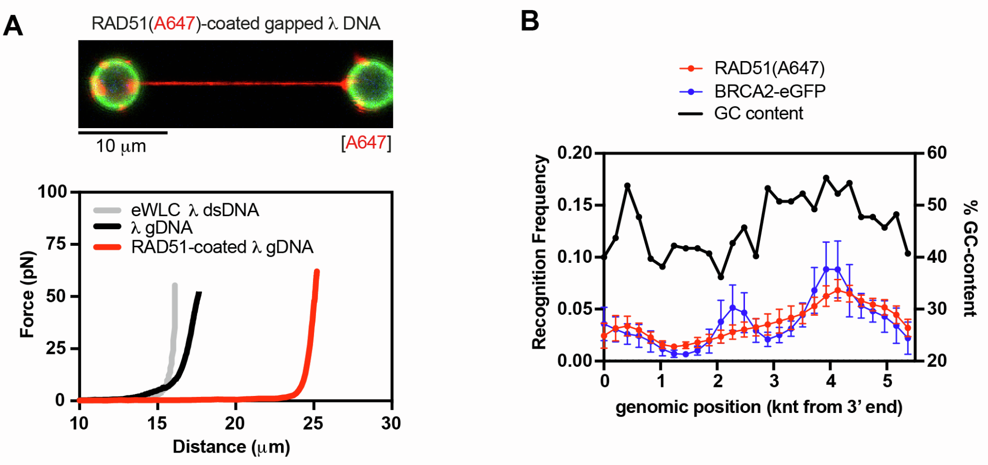

Figure S2: RAD51-gDNA characterization and further nucleation position-sequence analysis. Related to Fig. 2 and Fig. 3. (A) Top: representative image of RAD51(A647)-coated λ gDNA held at ~5 pN force. Bottom: force extension curves of λ gDNA (with 5.374 knt-long ssDNA gap, black), force extension curves of λ gDNA coated with RAD51(A647) (red). Gray line represents extensible worm-like chain model (eWLC) for 48.5 kb λ dsDNA as a reference. **(B)** Position analysis of RAD51(A647) (red) or BRCA2-eGFP (blue) binding along the length of 5.374 knt ssDNA gap in the absence of RPA. N = 6 molecules. Error bars represent S.E.M. 200 nt bins. GC-content of the DNA sequence within each bin is indicated in black.

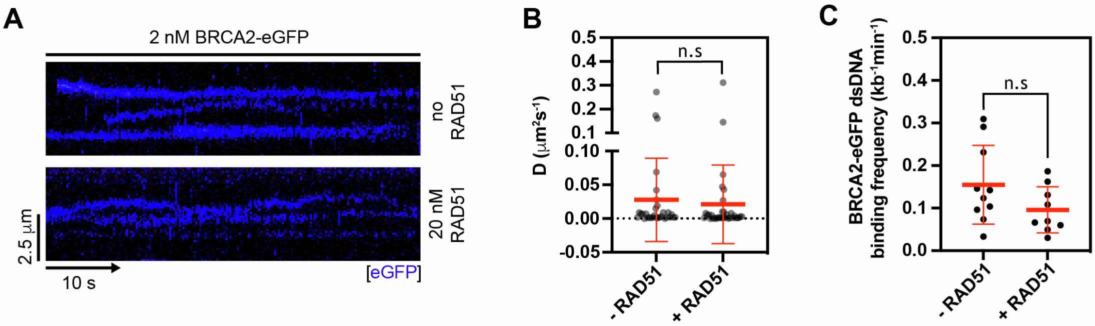

Figure S3: BRCA2 diffusion on dsDNA in the absence of RAD51. Related to Fig. 4. (A) Representative kymographs demonstrating diffusion of BRCA2-eGFP (blue) along λ dsDNA at 10 pN in the presence of 50 mM NaCl, 2 mM $MgCl_2$ and 2 mM ATP in the presence or absence of 20 nM RAD51 (unlabelled). **(B)** Diffusion coefficient (D) calculated from MSD analysis for BRCA2-eGFP complexes moving on dsDNA in the presence or absence of RAD51. Lines represent mean. Error bars represent S.D. P values by Mann-Whitney test. (n.s.) P > 0.05; (*) P ≤ 0.05; (**) P ≤ 0.01; (***) P ≤ 0.001; (****) P ≤ 0.0001. **(C)** dsDNA binding frequency of BRCA2-eGFP in the presence or absence of 20 nM RAD51, 50 mM NaCl, 2 mM $MgCl_2$, 2 mM ATP on dsDNA held at 10 pN force. Lines represent mean. Error bars represent S.D. P values by student t test.

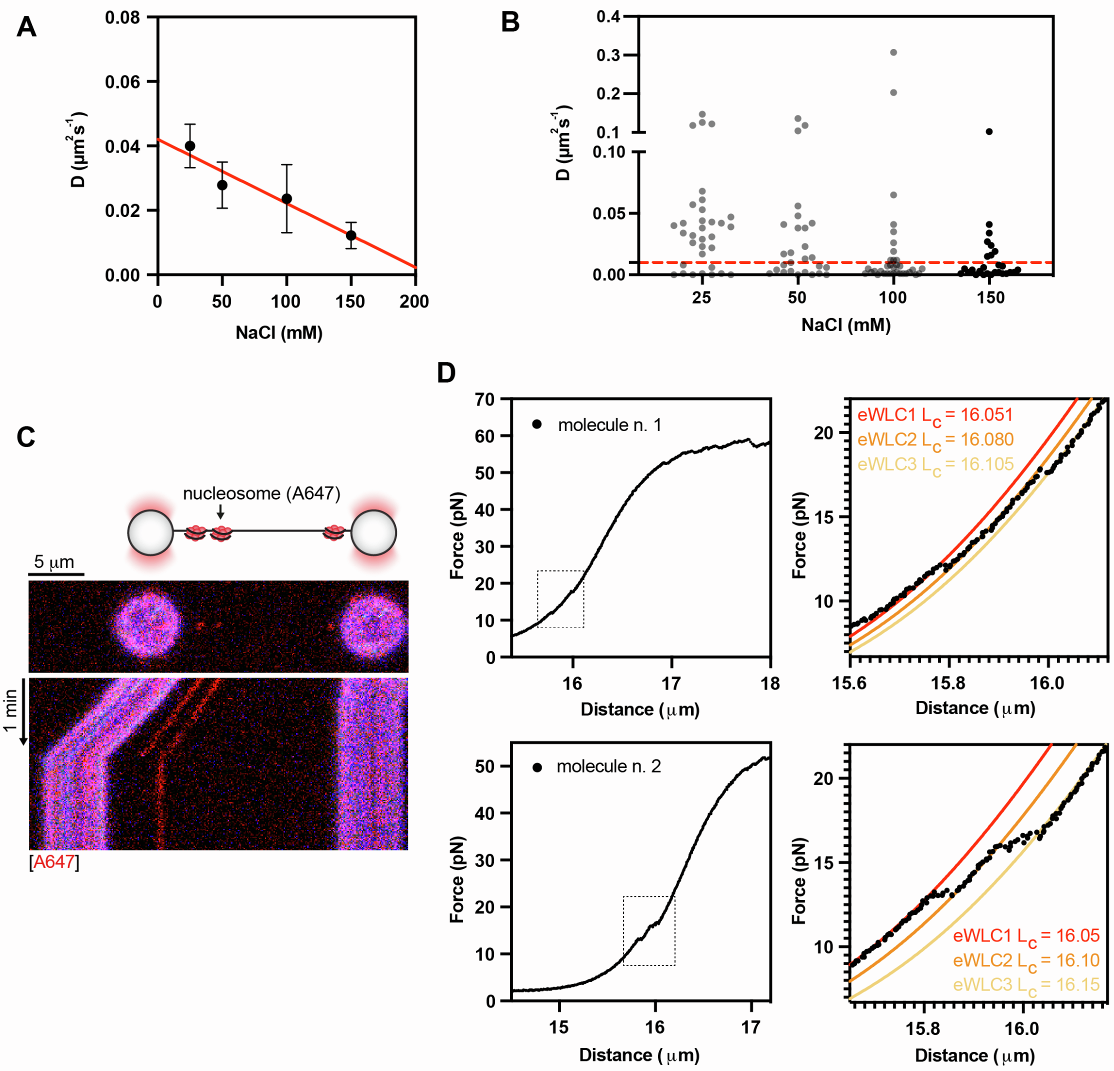

Figure S4: Characterization of chromatinized λ DNA. Related to Fig. 5. (A) Diffusion coefficient (N = 27-34) calculated for BRCA2-RAD51 complexes as a function of salt concentration. Error bars represent S.E.M**. (B)** Diffusion coefficient distribution calculated for BRCA2-RAD51 complexes at different salt concentrations. Red dashed line represents static fraction cut-off. **(C)** Representative kymograph showing stretching of sparsely chromatinized λ DNA. Alexa Fluor 647-labelled nucleosomes are shown in red. **(D)** Two examples of force-distance curve of sparsely chromatinized λ DNA. Individual unwrapping events characterized by sudden distance increase under the same force are highlighted in a dashed line box. Lines correspond to extensible worm-like chain (eWLC) models of dsDNA aligned with the corresponding force-extension curve segments. L_c corresponds to calculated contour length of DNA from the fit. L_c difference between adjacent eWLCs corresponds to length of DNA released during a nucleosome unwrapping event.

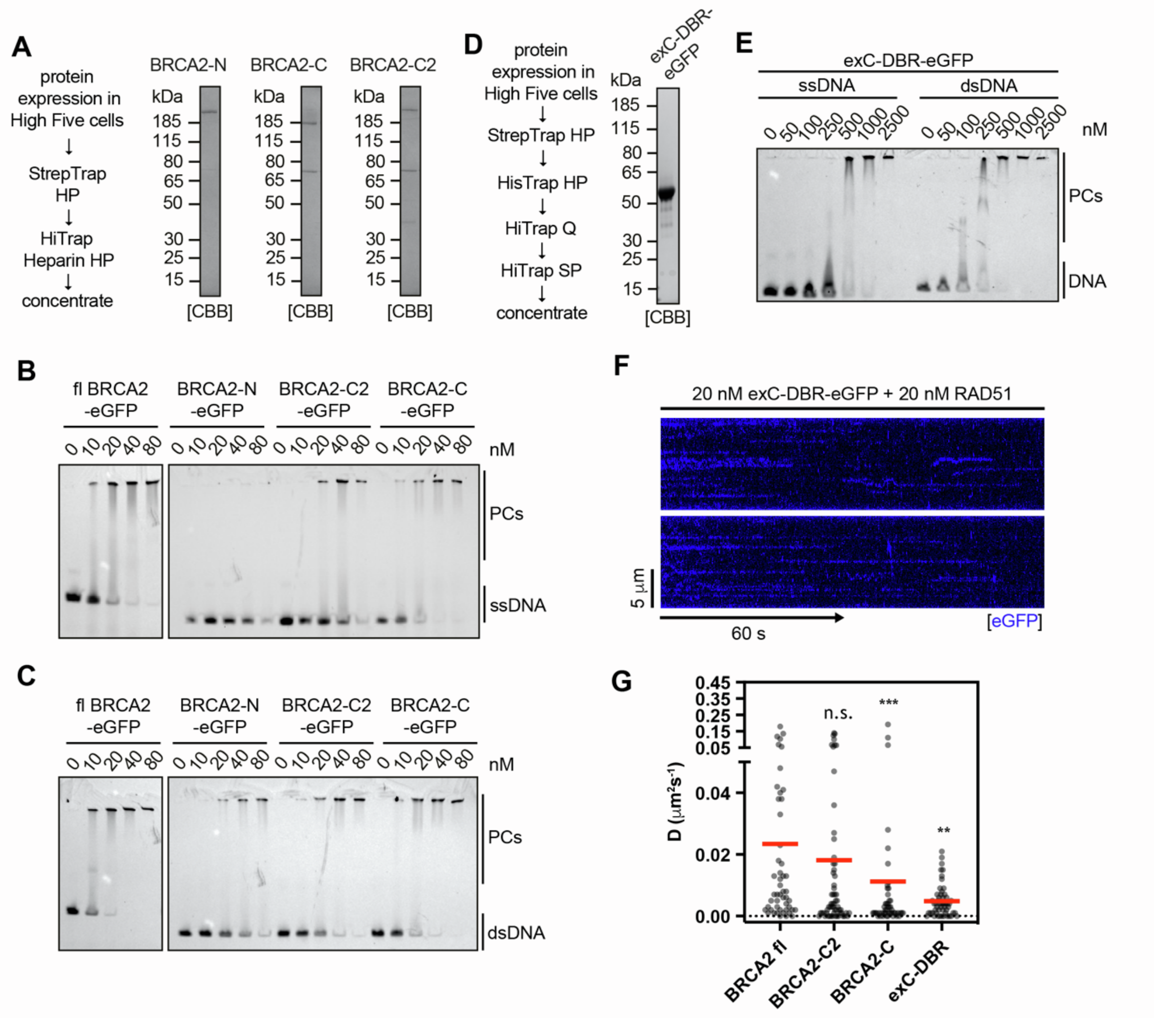

Figure S5: Further characterization of BRCA2 mutants. Related to Fig. 6. (A) Purification of BRCA2-N, BRCA2-C and BRCA2-C2 truncation mutants. Proteins were resolved by SDS-PAGE and stained by Coomasie Briliant Blue staining (CBB). **(B)** Electrophoretic mobility shift assay (EMSA) to assess affinity of BRCA2 and BRCA2 truncation mutants to 100mer ssDNA. **(C)** Electrophoretic mobility shift assay (EMSA) to assess affinity of BRCA2 and BRCA2 truncation mutants to 100mer dsDNA. PCs - protein-DNA complexes. **(D)** Purification of exC-DBR-eGFP fusion. Protein resolved by SDS-PAGE and stained by Coomasie Briliant Blue staining (CBB). **(E)** Electrophoretic mobility shift assay (EMSA) to assess affinity of exC-DBR-eGFP to 100mer ss- and dsDNA. PCs - protein-DNA complexes. **(F)** Two representative kymographs demonstrating limited diffusion of exC-DBR-eGFP (blue) along λ dsDNA at 10 pN in the presence of 50 mM NaCl, 2 mM $MgCl_2$, 2 mM ATP and 20 nM RAD51 (unlabelled). **(G)** Diffusion coefficient (D) calculated from MSD analysis for exC-DBR-eGFP molecules moving on dsDNA in the presence of RAD51. Lines represent mean. Data for BRCA2 fl, BRCA2-C and BRCA2-C2 are of the same dataset as in Fig. 6H. P values by Mann-Whitney test. (n.s.) $P > 0.05$; (*) $P \leq 0.05$; (**) $P \leq 0.01$; (***) $P \leq 0.001$; (****) $P \leq 0.0001$.

Oligonucleotides		
Oligonucleotide name and sequence	Source	Identifier
100mer ssDNA<br>5'-Cy5-<br>CCAAGAAGCTGTTCAGAATCAGAATGAGCCGCAACTT<br>CGGGATGAAAATGCTCACAATGACAAA-<br>TCTGTCCACGGAGTGCTTAATCCAACTTACCAAGCT-3'	IDT, this study	N/A
100mer dsDNA bottom<br>5'-<br>AGCTTGGTAAGTTGGATTAAGCACTCCGTGGACAGAT<br>TTGTCATTGTGAGCATTTTCATCCCGAAGTTGCGGCTC<br>ATTCTGATTCTGAACAGCTTCTTGG-3'	IDT, this study	N/A
3'tailed DNA top<br>5'-Cy5-<br>CTGCTTTATCAAGATAATTTTTCGACTCATCAGAAATA<br>TCCGTTTCCTATATTTATTCCTATTA-<br>TGTTTTATTCATTTACTTATTCTTTATGTTCATTTTTTAT<br>ATCCTTTACTTTATTTTCTCTGTTTATTCATTTACTTATT<br>TTGTATTATCCTTATCTTATTTA-3'	IDT, this study	N/A
3'tailed DNA bottom<br>5'-CGGATATTTCTGATGAGTCGAAAAATTATCTTGA-<br>TAAAGCAG-3'	IDT, this study	N/A
biot-SwaI-2023bp-f<br>5'-biotin-<br>AACAAAATATTAACGCTTACAATTTAAATGGTGGCAC<br>TTTTCG-3'	IDT, this study	N/A
2023bp-r<br>5'-TCAACGTGCAATCAAGTTAATGAATCGG-3'	IDT, this study	N/A
biot-SwaI-rc<br>5'-<br>CGAAAAGTGCCACCATTTAAATTGTAAGCGTTAATAT<br>TTTGTT-3'	IDT, this study	N/A
λ DNA end-cap 1<br>5'-<br>AGGTCGCCGCCCGGAGTTGAACG(BT)(BT)T(BT)T(BT)<br>ACGTTCAACTCC-3'	IDT, Belan et al, 2021	N/A
λ DNA end-cap 2<br>5'-GGGCGGCGA<br>CCTCAAGTTGGACAA(BT)T(BT)T(BT)(BT)TGTCCAACT<br>TG-3'	IDT, Belan et al, 2021	N/A
λ DNA handles oligo 1<br>5'-GGGCGGCGACCTGGACAA-3'	Sigma Aldrich, Belan et al, 2021	N/A
λ DNA handles oligo 2<br>5'- AGGTCGCCGCCCTTTTTTTT(BT)TT(BT)TT(BT)-3'	Sigma Aldrich, Belan et al, 2021	N/A

λ DNA handles oligo 3<br>5'-<br>T(BT)TT(BT)TT(BT)TTTTTTTAGAGTACTGTACGATCTA<br>GCATCAATCTTGTCC-3')	Sigma Aldrich, Belan et al, 2021	N/A
crRNA λ4<br>5'-CAGAUAUAGCCUGGUGGUUCGUUUUAGAGC<br>UAUGCUGUUUUG-3'	IDT, Belan et al, 2021	N/A
crRNA λ5<br>5'-<br>GGCAAUGCCGAUGGCGAUAGGUUUUAGAGCUAUG<br>CUGUUUUG-3'	IDT, Belan et al, 2021	N/A
tracr RNA (trRNA)<br>5'-GGACAGCAUAGCAAGU<br>UAAAAUAAGGCUAGUCCGUUAUCAACUUGAAA<br>AAGUGGCACCGAGUCGGUGCUUUUU-3'	IDT, Belan et al, 2021	N/A

Table S1: Oligonucleotides used in the study. Related to KEY RESOURCE TABLE.
